# Evolution-guided prioritization identifies a tissue-specific phosphorylation switch on herpes simplex virus 1 UL7 regulating viral replication and pathogenicity

**DOI:** 10.1128/jvi.00200-26

**Published:** 2026-04-30

**Authors:** Akihisa Kato, Takanori Tannaka, Ryoji Iwasaki, Saori Shio, Shaocong Liu, Kousuke Takeshima, Yuhei Maruzuru, Yasushi Kawaguchi

**Affiliations:** 1 Division of Molecular Virology, Department of Microbiology and Immunology, The Institute of Medical Science, The University of Tokyo, Tokyo, Japan; 2 Department of Infectious Disease Control, International Research Center for Infectious Diseases, The Institute of Medical Science, The University of Tokyo, Tokyo, Japan; 3 Research Center for Asian Infectious Diseases, The Institute of Medical Science, The University of Tokyo, Tokyo, Japan; 4 PRESTO, Japan Science and Technology Agency (JST) 13501, Kawaguchi, Japan; 5 Pandemic Preparedness, Infection and Advanced Research Center, The University of Tokyo, Tokyo, Japan; University of Virginia, Charlottesville, Virginia, USA

**Keywords:** evolution-guided prioritization, HSV-1, UL7, phosphorylation, pathogenicity

## Abstract

Although numerous phosphorylation sites on herpes simplex virus 1 (HSV-1) proteins have been identified through phosphoproteomic analyses, their virological significance remains largely unclear. Here, we developed an evolution-guided prioritization strategy that integrates phosphoproteomic data with residue-level conservation across the genus *Simplexvirus* to identify phosphorylation sites as candidates for functional relevance. Applying this strategy to a previously reported phosphoproteomic data set, we identified 46 phosphorylation sites with a conservation level of 88% or higher. Among them, we focused on two phosphorylation sites, UL6 Tyr-234 and UL7 Tyr-89, which are conserved in 100% and 88% of *Simplexvirus* species, respectively. Phosphomimetic mutations at either site significantly reduced progeny virus yields in cultured cells, suggesting that phosphorylation at these sites can function as an inhibitory switch regulating HSV-1 replication. In agreement with this, in-depth analyses of phosphorylation at UL7 Tyr-89 during HSV-1 infection revealed that the phosphomimetic mutation led to phenotypes similar to those of the UL7 null mutation in virion morphogenesis, HSV-1 replication in the central nervous system (CNS) and eyes of mice, as well as CNS pathogenicity and ocular pathogenic manifestations. Notably, the non-phosphorylatable mutation at this site had little effect on HSV-1 infection in cultured cells, whereas it significantly reduced HSV-1 replication and pathogenicity in the CNS, but not in the eyes of mice. These results suggest that our strategy effectively prioritizes phosphorylation sites for functional investigation and that phosphorylation at UL7 Tyr-89 serves as a context-dependent inhibitory switch, fine-tuning UL7 activity in a tissue-specific manner, particularly in the CNS.

**IMPORTANCE:**

Intricate phosphorylation-dependent regulatory mechanisms enable viruses to diversify the functions of their proteins, profoundly shaping viral replication and pathogenicity. Although phosphoproteomic analyses have produced an expanding catalog of phosphorylation sites on viral proteins, a considerable proportion of these modifications are likely non-functional. This creates a pressing need for a prioritization strategy to predict functionally relevant phosphorylation sites. To address this, we developed an evolution-guided prioritization strategy that integrates phosphoproteomic data with genus-level conservation. Using this strategy, we prioritized two tyrosine phosphorylation sites for functional analysis. Notably, phosphorylation at one of these sites, UL7 Tyr-89, functions as a tissue-specific regulatory mechanism that fine-tunes UL7 activity and modulates herpes simplex virus 1 replication and pathogenicity in the central nervous system. This strategy provides a practical framework for prioritizing candidate regulatory sites for functional investigation in viral replication and pathogenicity.

## INTRODUCTION

Phosphorylation of cellular proteins is a pivotal post-translational modification that regulates not only protein activity but also protein stability, conformation, subcellular localization, interactions with binding partners, and degradation dynamics (1). Similarly, a wide range of viral proteins are also subject to phosphorylation in infected cells (2–5), and it is widely believed that viruses exploit intricate phosphorylation-dependent regulatory mechanisms to expand the functional diversity of their proteins, thereby playing crucial roles in viral replication and pathogenicity. Indeed, accumulating evidence demonstrates that phosphorylation of diverse viral proteins, including viral enzymes, structural components, and regulatory factors, modulates multiple aspects of viral infection (6, 7). Therefore, elucidating the virological significance of viral protein phosphorylation, as well as the underlying regulatory mechanisms of these phosphorylation events, is essential for understanding the molecular basis of viral replication and pathogenesis. The initial steps of such studies are to identify phosphorylation sites in viral proteins and elucidate the functional significance of phosphorylation at these sites.

High-resolution mass spectrometry (MS)-based phosphoproteomics is a powerful approach for mapping phosphorylation sites (8). Indeed, MS-based phosphoproteomic analyses of cultured cells infected with herpes simplex virus 1 (HSV-1), an extensively studied herpesvirus, cataloged several hundred phosphorylation sites on HSV-1 proteins (3, 4, 9). Although several phosphorylation events at the sites identified by this technique have been linked to HSV-1 replication and/or pathogenicity (3, 9, 10), it is assumed that these catalogs contain a considerable number of sites whose phosphorylation is unlikely to influence target protein function. Indeed, several phosphorylation sites identified by this method have also been reported not to be involved in HSV-1 replication or pathogenicity (11). Because investigating the functional significance of phosphorylation is often experimentally demanding and resource-intensive, establishing a prioritization strategy to predict functionally relevant phosphorylation sites would provide an effective framework aimed at facilitating the elucidation of the molecular basis of viral infection based on viral protein phosphorylation.

Evolutionarily conserved regulatory sites and domains have often been used as indicators to predict functionally important phosphorylation sites (12, 13). Viruses in the family *Herpesviridae* are subclassified into three subfamilies: *Alphaherpesvirinae*, *Betaherpesvirinae*, and *Gammaherpesvirinae*, and HSV-1 belongs to the genus *Simplexvirus* within the *Alphaherpesvirinae* subfamily (14). To predict functionally relevant phosphorylation sites in HSV-1, assessing their evolutionary conservation across all herpesviruses would be ideal. However, nearly half of HSV-1 genes are not conserved across the three subfamilies (14). Moreover, genetic diversity among herpesvirus genes increases substantially at higher taxonomic levels, including genus, subfamily, and family. At the genus level, viral gene sets are well conserved, and conserved sites can be readily identified using amino-acid sequence alignments (15, 16). In contrast, at higher taxonomic levels, some viral genes are absent, and reliable identification of conserved sites often requires structure-based comparisons (17, 18). Therefore, we consider that comparative analyses at the genus level are most appropriate for accurately identifying the maximum number of evolutionarily conserved phosphorylation sites based on amino acid sequence alignments. In this study, we developed an evolution-guided framework that integrates genus-level conservation with phosphoproteomic data to systematically refine large-scale phosphoproteomic data sets and prioritize phylogenetically conserved phosphorylation sites with high potential for functional relevance. Using this framework, we focused on phosphorylation sites (Tyr-234 and Tyr-89) predicted in two distinct HSV-1 proteins, UL6 and UL7, respectively. Both proteins are conserved across the family *Herpesviridae*: UL6 functions as the portal protein required for DNA translocation into the capsid, whereas UL7 is a tegument protein incorporated into virions and has been implicated in secondary envelopment and cell-to-cell spread (19–22). We demonstrate that phosphomimetic mutations at UL6 Tyr-234 and UL7 Tyr-89 significantly reduce viral replication and plaque size in cultured cells. Notably, whereas the phosphomimetic mutation at UL7 Tyr-89 significantly reduces viral replication and pathogenicity in mice following both intracranial and ocular infections, the non-phosphorylatable mutation at this site affects viral replication and pathogenicity in mice following intracranial infection but not ocular infection. These findings raise the possibility that phosphorylation at UL7 Tyr-89 functions as a context-dependent inhibitory switch that fine-tunes UL7 activity in a tissue-specific manner, particularly within the central nervous system (CNS).

## RESULTS

### Conservation of phosphorylation sites on HSV-1 proteins across the genus *Simplexvirus*

Based on the phosphoproteomic data from HSV-1(17) syn^+^-infected human foreskin fibroblasts reported by Kulej et al. (4), we focused on 470 phosphorylation sites with greater than 75% probability of accurate phosphosite localization within their respective peptides, mapping to 63 HSV-1 proteins. Among these, 463 phosphorylation sites on 63 HSV-1 proteins were found to be conserved in the HSV-1(F) strain, which was used in this study. Seventeen viruses belonging to the genus *Simplexvirus* are classified by the International Committee on Taxonomy of Viruses (ICTV) (23). The coding sequence annotation information for these viruses, deposited in the NCBI Nucleotide database (Table S1), shows that 51 of the 63 HSV-1 proteins are annotated across all 17 viruses (Table S2). The remaining HSV-1 proteins were less widely annotated: UL31, UL45, and UL49 in 16; UL3 in 14; UL56 in 13; UL43 in 12; Us8A in 11; Us10, Us11, and ICP34.5 in 10; Us9 in 9; and Us12 in 8 of the 17 viruses (Table S2). Multiple alignments of the amino acid sequences of each of the 63 HSV-1 proteins and the annotated orthologs in the other 16 viruses of the genus *Simplexvirus* revealed that, among the 463 identified HSV-1 phosphorylation sites, 26 sites (5.6%) are conserved across all of the other 16 viruses, while 33 sites (7.1%) and 57 sites (12%) were conserved in at least 90% and 80% of the other viruses where orthologs were available, respectively (Fig. S1; Table S2). The remaining 406 sites were conserved in fewer than 80% of the other viruses with annotated orthologs (Fig. S1; Table S2). The highly conserved phosphorylation sites may therefore serve as candidate sites with high potential for functional relevance.

**Fig 1:**
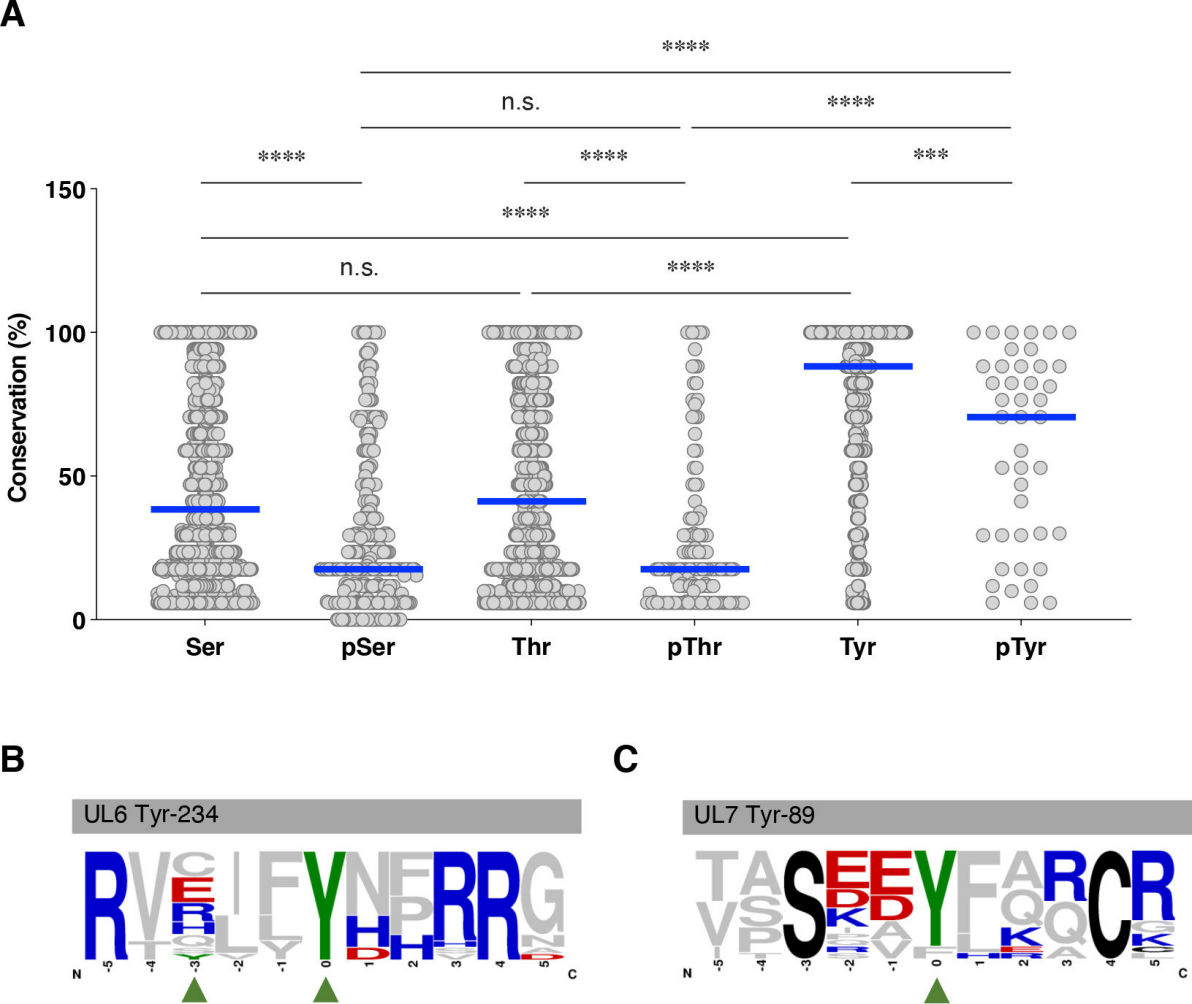
Conservation of UL6 and UL7 phosphorylation sites across the genus *Simplexvirus*. (**A**) Conservation percentages of serine (Ser), phosphorylated serine (pSer), threonine (Thr), phosphorylated threonine (pThr), tyrosine (Tyr), and phosphorylated tyrosine (pTyr) residues identified in HSV-1 (group sizes: Ser = 2,329, pSer = 282, Thr = 2,249, pThr = 138,Tyr = 955, and pTyr = 43) were shown across members of the genus *Simplexvirus*. Each data point represents an individual residue; blue bars indicate the median. Statistical significance was determined using Welch’s one-way ANOVA followed by pairwise Welch’s *t*-tests with Benjamini–Hochberg false discovery rate correction. *, *P* < 0.05; ***, *P* < 0.001; ****, *P* < 0.0001; and n.s., not significant. (**B and C**) WebLogo representation of UL6 (**B**) and UL7 (**C**) homologs among members of the genus *Simplexvirus*. The tyrosine phosphorylation sites identified by phosphoproteome analysis of HSV-1-infected human foreskin fibroblasts (4), UL6 Tyr-231, UL6 Tyr-234, and UL7 Tyr-89 in HSV-1, are highlighted in green and indicated by green arrowheads. Basic and acidic amino acids are shown in blue and red, respectively, whereas other fully conserved amino acids are shown in black.

We note that the median conservation percentage of tyrosine phosphorylation sites is significantly higher, being 3.0- and 4.0-fold greater than those of serine and threonine phosphorylation sites, respectively (Fig. 1A). In contrast, the median conservation percentage of tyrosine residues is only 2.3- and 2.1-fold higher than those of serine and threonine residues, respectively (Fig. 1A). These results suggest that tyrosine phosphorylation tends to occur at more evolutionarily conserved residues, rather than merely reflecting overall residue conservation in the genus *Simplexvirus*, highlighting the potential functional importance of tyrosine phosphorylation in these viruses. However, information on the functional significance of tyrosine phosphorylation during HSV-1 infection remains limited.

Given the limited functional characterization of tyrosine phosphorylation despite its strong evolutionary conservation, we focused on three tyrosine phosphorylation sites, UL6 Tyr-231, UL6 Tyr-234, and UL7 Tyr-89 (Fig. 1B and C), to evaluate the utility of our prioritization strategy for predicting functionally relevant phosphorylation sites. We selected these sites based on their varying degrees of evolutionary conservation (5.9%, 100%, and 88%, respectively) across the genus *Simplexvirus* (Fig. 1B and C; Table S2). In particular, UL6 contains both a perfectly conserved tyrosine phosphorylation site (Tyr-234) and a poorly conserved site (Tyr-231), allowing us to examine how evolutionary conservation relates to functional relevance (Fig. 1B; Table S2). In addition, previous studies reported that tyrosine phosphorylation of gB at an 88% conserved site (Tyr-889) is functionally important (4, 24), which prompted us to similarly investigate the UL7 Tyr-89 site, also conserved in 88% of sequences (Fig. 1C; Table S2). We then investigated the functional significance of their phosphorylation during HSV-1 infection in cultured cells.

### Construction of recombinant viruses

**Fig 2:**
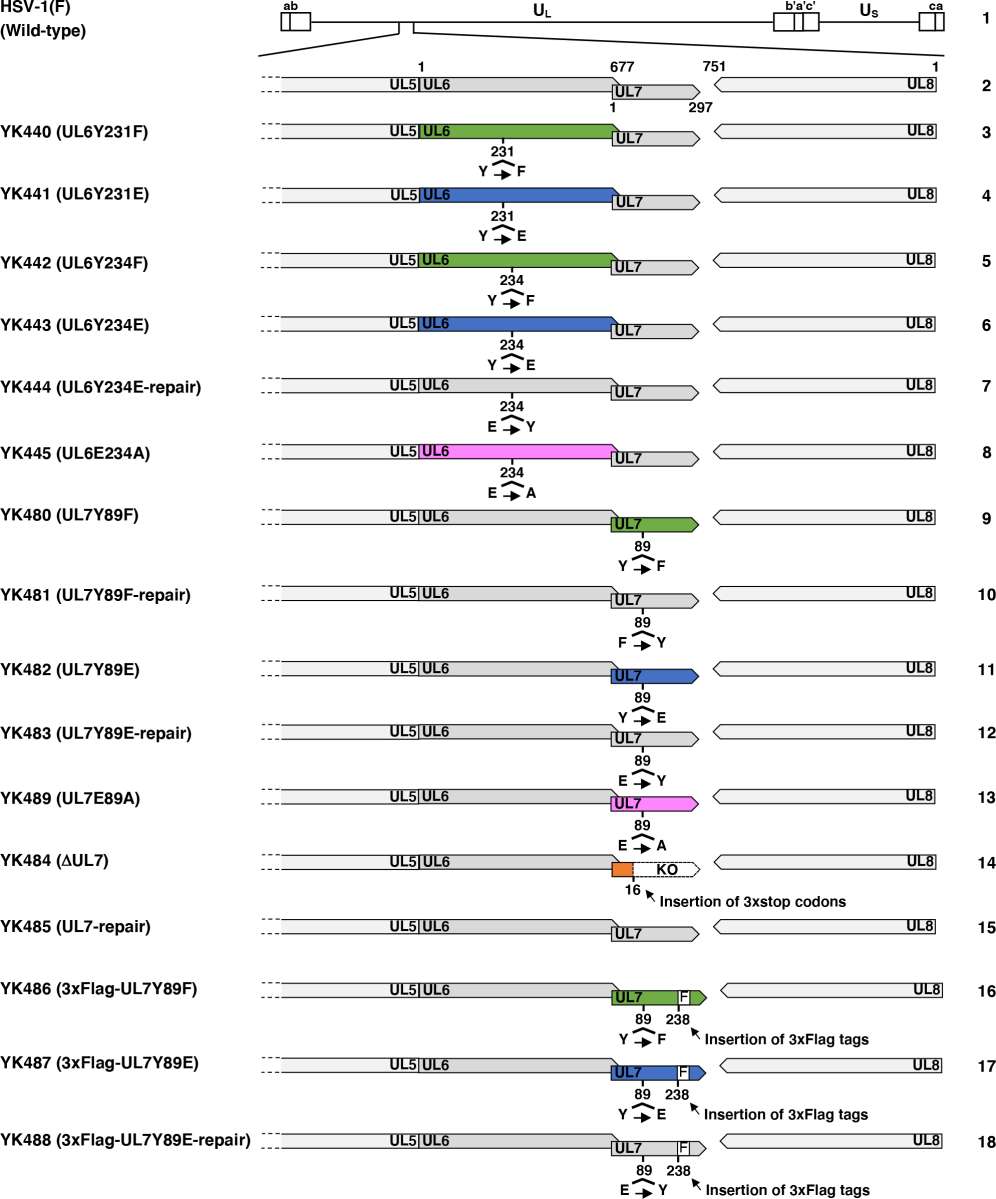
Schematic diagrams of the genome structure of HSV-1(F) and recombinant viruses used in this study. Line 1, wild-type HSV-1(F) genome; line 2, the domains of the UL5 to UL8 genes; lines 4–8, recombinant viruses with mutations in the UL6 genes; lines 9–15, recombinant viruses with mutations in the UL7 genes; and lines 16–18, recombinant viruses carrying 3xFlag-tagged UL7 with mutations in the UL7 genes. The 3xFlag tag sequence is represented as “F.”

**Fig 3:**
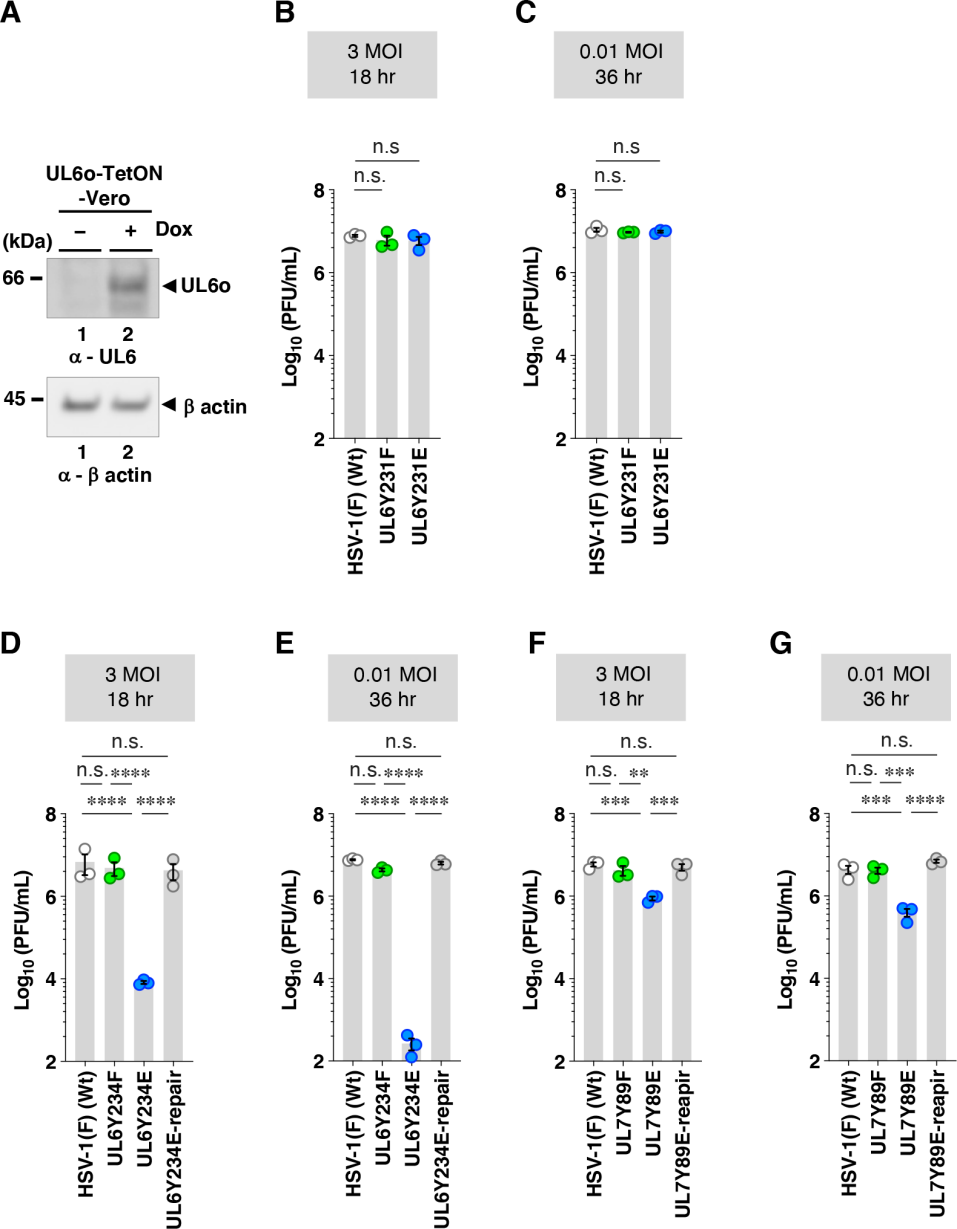
Effects of non-phosphorylatable or constitutive phosphomimetic mutations in UL6 and UL7 on viral replication in Vero cells. (**A**) UL6o-TetON-Vero cells were mock-treated or treated with doxycycline (Dox) (1 mg/mL), harvested 24 h post-treatment, and analyzed by immunoblotting with antibodies to UL6 and β actin. (**B–G**) Vero cells were infected with wild-type HSV-1(F) (**B–G**), YK440 (UL6 Y231F) (**B and C**), YK441 (UL6 Y231E) (**B and C**), YK442 (UL6 Y234F) (**D and E**), YK443 (UL6 Y234E) (**D and E**), YK444 (UL6 Y234E-repair) (**D and E**), YK480 (UL7 Y89F) (**F and G**), YK482 (UL7 Y89E) (**F and G**), and YK483 (UL7 Y89E-repair) (**F and G**) at MOIs of 3 or 0.01. Total virus from cell culture supernatants and infected cells was harvested at 18 h (**B, D, and F**) or 36 h (**C, E, and G**) post-infection and assayed on UL6o-TetON-Vero cells (**B–E**) and Vero cells (**F and G**). Each data point represents the mean ± SEM of the results of three independent experiments. Statistical significance was assessed by one-way ANOVA followed by Tukey’s test. **, *P* < 0.01; ***, *P* < 0.001; ****, *P* < 0.0001; and n.s., not significant.

To examine the effects of phosphorylation at UL7 Tyr-89, UL6 Tyr-234, and UL6 Tyr-231 during HSV-1 infection, we generated recombinant viruses in which each tyrosine residue was replaced either with phenylalanine to abolish phosphorylation or with glutamic acid to mimic it (25, 26) (Fig. 2). Specifically, we constructed the following recombinant viruses: YK440 (UL6Y231F) and YK441 (UL6Y231E), in which Tyr-231 in UL6 was replaced with phenylalanine or glutamic acid, respectively; YK442 (UL6Y234F) and YK443 (UL6Y234E), with the same substitutions at Tyr-234 in UL6; and YK480 (UL7Y89F) and YK482 (UL7Y89E), with the same substitutions at Tyr-89 in UL7 (Fig. 2). As a control, we constructed a UL7 null mutant virus YK484 (ΔUL7), in which the UL7 gene was disrupted by the insertion of three stop codons, as previously described (22) (Fig. 2). In addition, we constructed repaired versions of YK443 (UL6Y234E), YK480 (UL7Y89F), YK482 (UL7Y89E), and YK484 (ΔUL7), designated as YK444 (UL6Y234E-repair), YK481 (UL7Y89F-repair), YK483 (UL7Y89E-repair), and YK485 (ΔUL7-repair), respectively, (Fig. 2). In preliminary experiments, YK443 (UL6Y234E) could not be reconstituted by transfecting its infectious genome clone, generated in *Escherichia coli*, into Vero cells permissive for HSV-1 infection, suggesting that the Y234E mutation in UL6 impairs HSV-1 replication in cultured cells. Therefore, we established UL6o-TetON-Vero cells, in which UL6 expression is induced by the addition of doxycycline, to support the replication of YK443 (UL6Y234E) (Fig. 3A). These cells were used to reconstitute YK443 (UL6Y234E), and all viruses used for comparative analyses with YK443 (UL6Y234E) in this study were propagated and titrated using UL6o-TetON-Vero cells.

### Effects of phosphorylation at UL6 Tyr-231, UL6 Tyr-234, and UL7 Tyr-89 on HSV-1 progeny virus yields and plaque sizes in cultured cells

**Fig 4:**
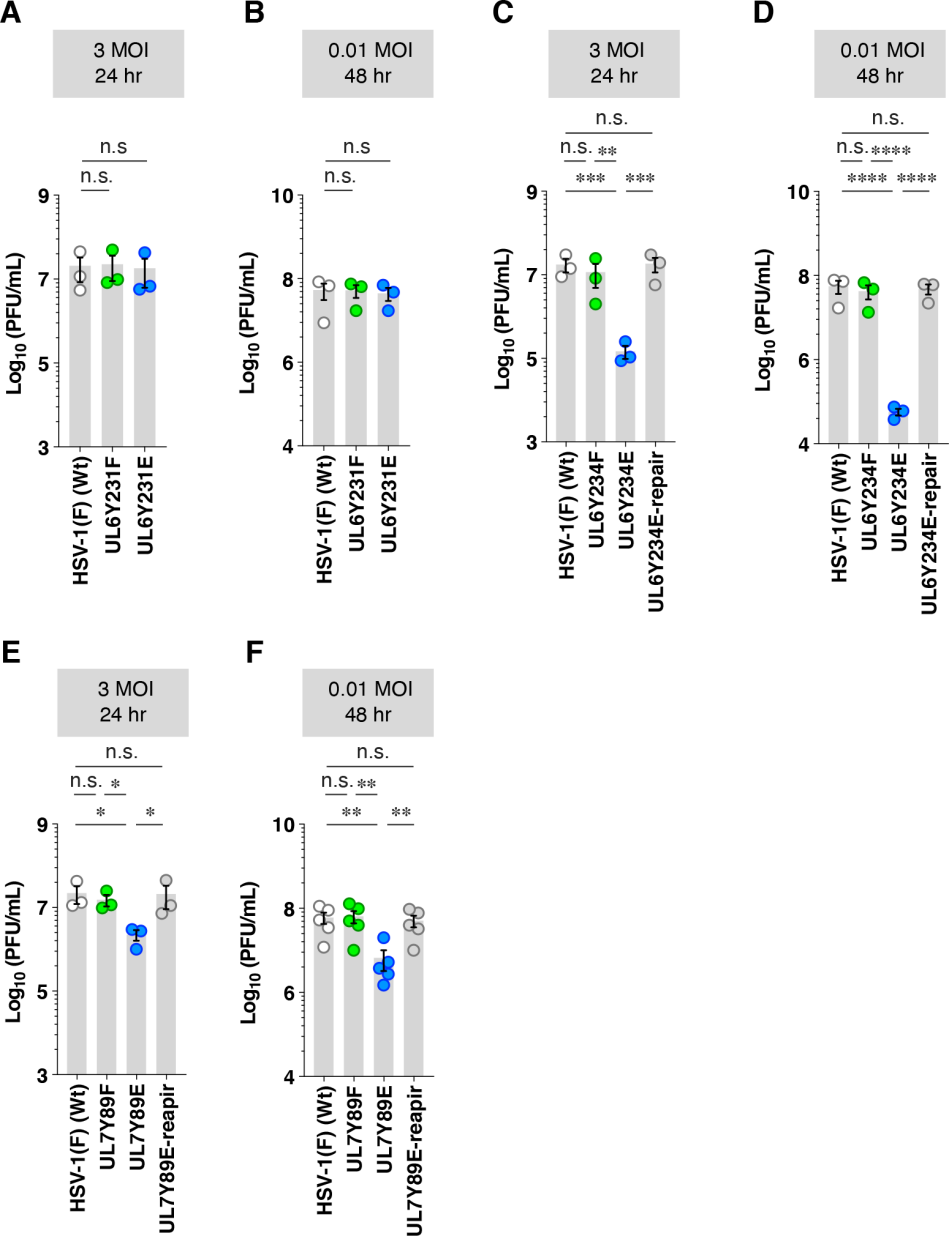
Effects of non-phosphorylatable or constitutive phosphomimetic mutations in UL6 and UL7 on viral replication in ARPE-19 cells. (**A–F**) ARPE-19 cells were infected with wild-type HSV-1(F) (**A–F**), YK440 (UL6 Y231F) (**A and B**), YK441 (UL6 Y231E) (**A and B**), YK442 (UL6 Y234F) (**C and D**), YK443 (UL6 Y234E) (**C and D**), YK444 (UL6 Y234E-repair) (**C and D**), YK480 (UL7 Y89F) (**E and F**), YK482 (UL7 Y89E) (**E and F**), and YK483 (UL7 Y89E-repair) (**E and F**) at MOIs of 3 or 0.01. Total virus from cell culture supernatants and infected cells was harvested at 24 h (**A, C, and E**) or 48 h (**C, E, and G**) post-infection and assayed on UL6o-TetON-Vero cells (**A–D**) and Vero cells (**E and F**). Each data point represents the mean ± SEM of the results of three (**A–E**) or five (**F**) independent experiments. Statistical significance was assessed by one-way ANOVA followed by Tukey’s test. *, *P* < 0.05; **, *P* < 0.01; ***, *P* < 0.001; ****, *P* < 0.0001; and n.s., not significant.

To investigate the effects of phosphorylation at the tyrosine phosphorylation sites in UL6 and UL7 during HSV-1 infection in cultured cells, we examined the effects of non-phosphorylatable and phosphomimetic mutations at the phosphorylation sites on HSV-1 progeny virus yields in Vero cells. Vero cells were infected with wild-type HSV-1(F), YK440 (UL6Y231F), YK441 (UL6Y231E), YK442 (UL6Y234F), YK443 (UL6Y234E), YK444 (UL6Y234E-repair), YK480 (UL7Y89F), YK482 (UL7Y89E), or YK483 (UL7Y89E-repair) at multiplicities of infection (MOIs) of 3 or 0.01. Viral titers were determined at 18 h post-infection for an MOI of 3, and at 36 h post-infection for an MOI of 0.01. Progeny virus yields of YK440 (UL6Y231F) and YK441 (UL6Y231E) were comparable to those of wild-type HSV-1(F) at both MOIs (Fig. 3B and C), indicating that mutations at UL6 Tyr-231 have no appreciable effect on HSV-1 replication in Vero cells. In contrast, progeny virus yields of YK443 (UL6Y234E) were significantly lower than those of wild-type HSV-1(F) and YK444 (UL6Y234E-repair) at both MOIs (Fig. 3D and E). Similarly, YK482 (UL7Y89E) showed significantly reduced progeny virus yields compared to wild-type HSV-1(F) and YK483 (UL7Y89E-repair) at both MOIs (Fig. 3F and G), whereas YK442 (UL6Y234F) and YK480 (UL7Y89F) yielded virus titers comparable to wild-type HSV-1(F) (Fig. 3D through G). Comparable trends were also observed in ARPE-19 cells, which possess intact innate immune responses and represent a more physiological human epithelial cell line (Fig. 4) (27, 28).

**Fig 5:**
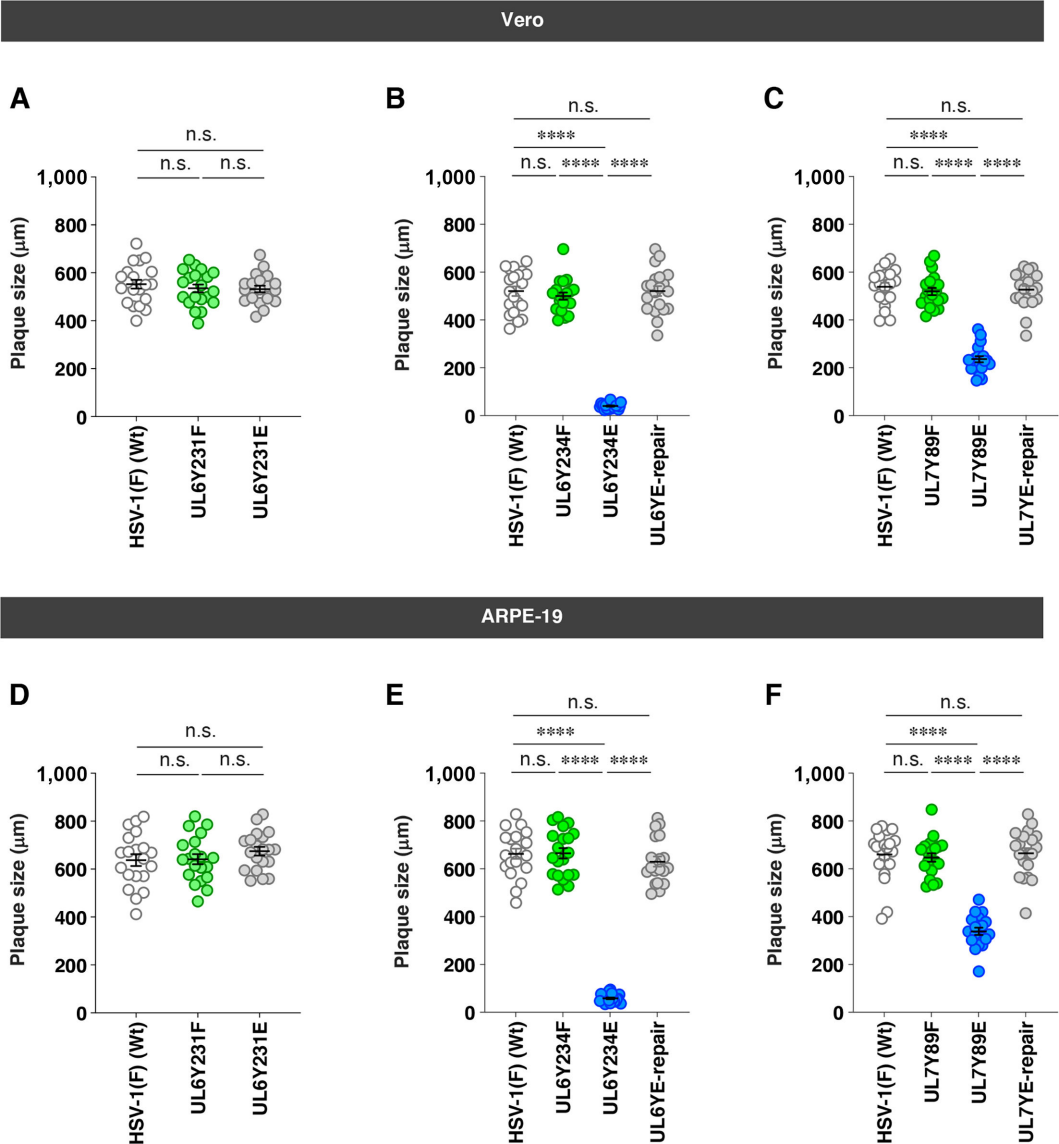
Effects of non-phosphorylatable or constitutive phosphomimetic mutations in UL6 and UL7 on plaque size in Vero and ARPE-19 cells. (**A–F**) Vero (**A–C**) and ARPE-19 (**D–F**) cells were infected with wild-type HSV-1(F) (**A–F**), YK440 (UL6 Y231F) (**A and D**), YK441 (UL6 Y231E) (**A and D**), YK442 (UL6 Y234F) (**B and E**), YK443 (UL6 Y234E) (**B and E**), YK444 (UL6 Y234E-repair) (**B and E**), YK480 (UL7 Y89F) (**C and F**), YK482 (UL7 Y89E) (**C and F**), and YK483 (UL7 Y89E-repair) (**C and F**) under plaque assay conditions. The diameters of 20 single plaques for each of the indicated viruses were measured 2 days post-infection. Each point represents a single plaque size. The horizontal line and error bars indicate the mean ± SEM for each group. Statistical significance was assessed by one-way ANOVA followed by Tukey’s test. ****, *P* < 0.0001 and n.s., not significant.

In agreement with the observed progeny virus yields, YK440 (UL6Y231F) and YK441 (UL6Y231E) produced plaques comparable in size to those of wild-type HSV-1(F) in Vero and ARPE-19 cells (Fig. 5A and D). YK443 (UL6Y234E) and YK482 (UL7Y89E) produced markedly smaller plaques than wild-type HSV-1(F), YK444 (UL6Y234E-repair), and YK483 (UL7Y89E-repair), whereas YK442 (UL6Y234F) and YK480 (UL7Y89F) formed plaques comparable in size to those of wild-type HSV-1(F) (Fig. 5B, C, E, and F).

**Fig 6:**
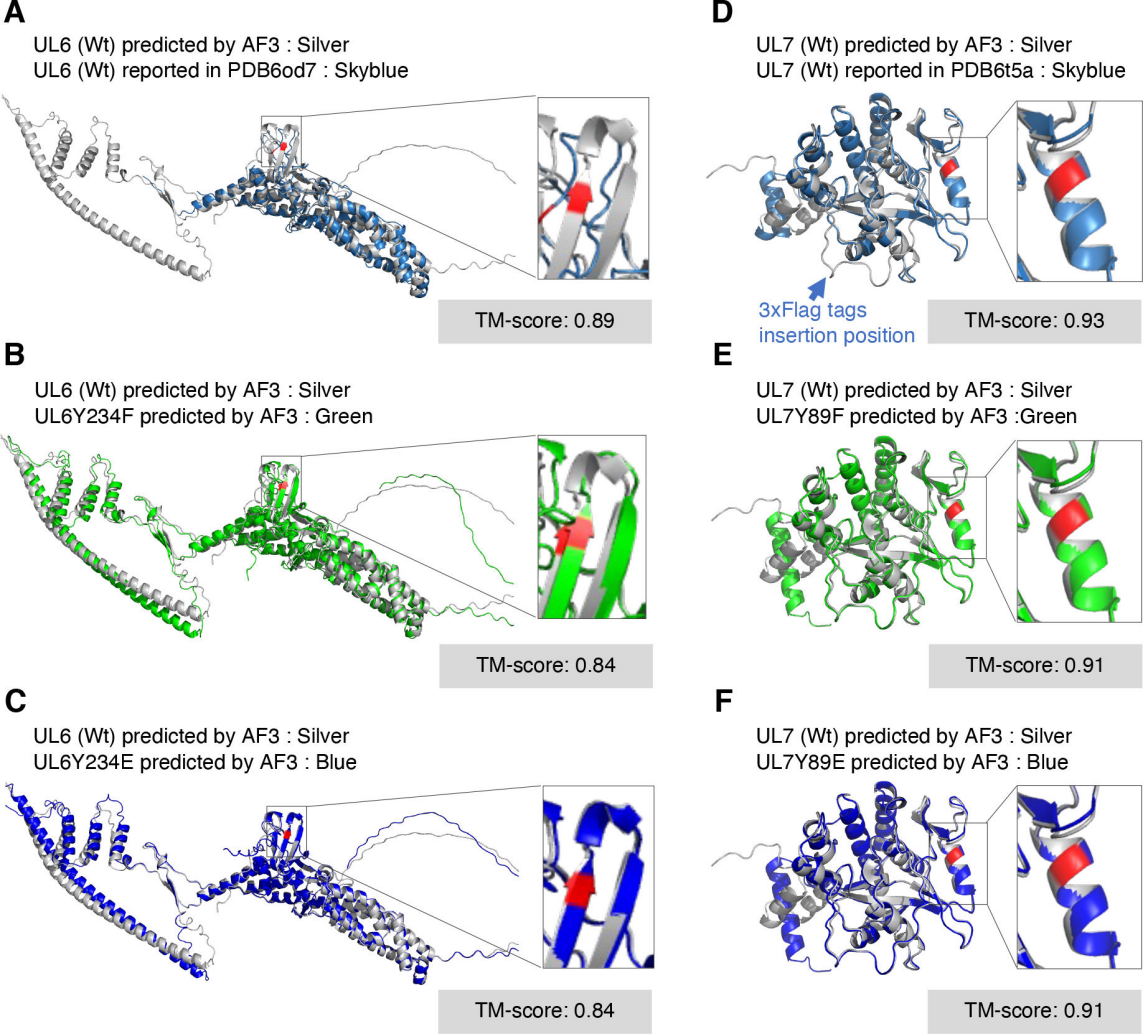
Predicted structural comparisons between wild-type and non-phosphorylatable or phosphomimetic mutants of HSV-1 UL6 and UL7. (**A**) Overlay of the predicted full-length wild-type HSV-1(F) UL6 structure modeled by AlphaFold3 (silver) and the Cryo-EM structure of wild-type HSV-1(KOS) UL6 (amino acids 33–306; PDB ID: 6od7) (sky blue). (**B and C**) Overlay of the predicted full-length wild-type HSV-1(F) UL6 structure (silver) with the predicted structures of HSV-1(F) UL6 carrying the Y234F (green) (**B**) or Y234E (blue) (**C**) mutations, modeled by AlphaFold3. (**D**) Overlay of the predicted full-length wild-type HSV-1(F) UL7 structure modeled by AlphaFold3 (silver) and the Cryo-EM structure of wild-type HSV-1(KOS) UL7 (amino acids 11–234; PDB ID: 6t5a) (sky blue). (**E and F**) Overlay of the predicted full-length wild-type HSV-1(F) UL7 structure (silver) with the predicted structures of HSV-1(F) UL7 carrying the Y89F (green) (**E**) or Y89E (blue) (**F**) mutations, modeled by AlphaFold3. The substituted residues and their wild-type counterparts (UL6 Tyr-234 and UL7 Tyr-89) are indicated in red, and zoomed-in views of the modified regions are shown to facilitate evaluation of potential local conformational changes. The TM scores (29) for each structural comparison are also indicated.

**Fig 7:**
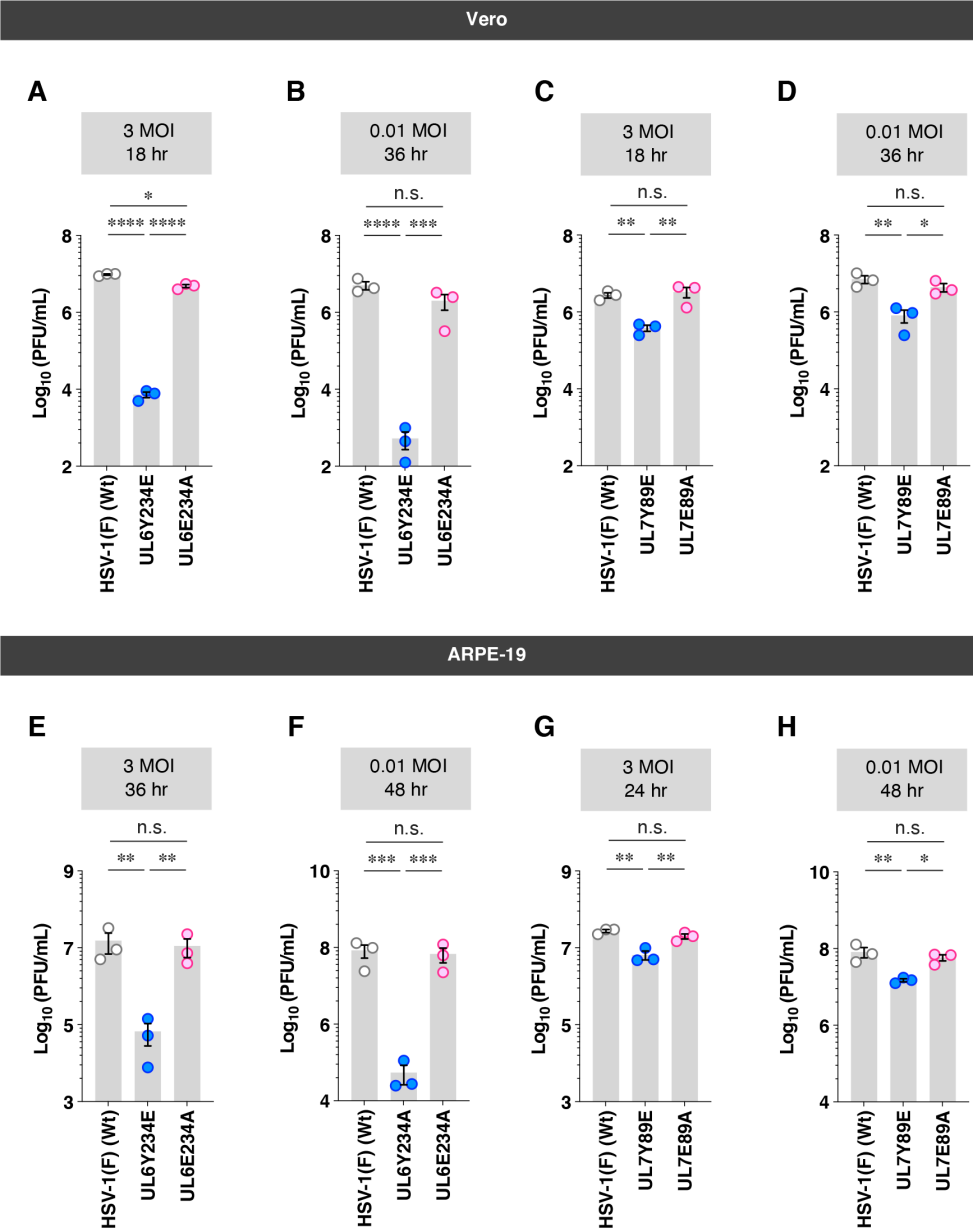
Effects of alanine substitution at UL6 and UL7 phosphorylation sites on viral replication in Vero and ARPE-19 cells. (**A–H**) Vero (**A–D**) and ARPE-19 (**E–H**) cells were infected with wild-type HSV-1(F) (**A–H**), YK443 (UL6 Y234E) (**A, B, E, and F**), YK445 (UL6 E234A) (**A, B, E, and F**), YK482 (UL7 Y89E) (**C, D, G, and H**), and YK489 (UL7 E89A) (**C, D, G, and H**) at MOIs of 3 or 0.01. Total virus from cell culture supernatants and infected cells was harvested at 18 h (**A and C**), 24 h (**E and G**), 36 h (**B and D**), or 48 h (**F and H**) post-infection and assayed on UL6o-TetON-Vero cells (**A, B, E, and F**) and Vero cells (**C, D, G, and H**). Each data point represents the mean ± SEM of the results of three independent experiments. Statistical significance was assessed by one-way ANOVA followed by Tukey’s test. *, *P* < 0.05; **, *P* < 0.01; ***, *P* < 0.001; ****, *P* < 0.0001; and n.s., not significant.

**Fig 8:**
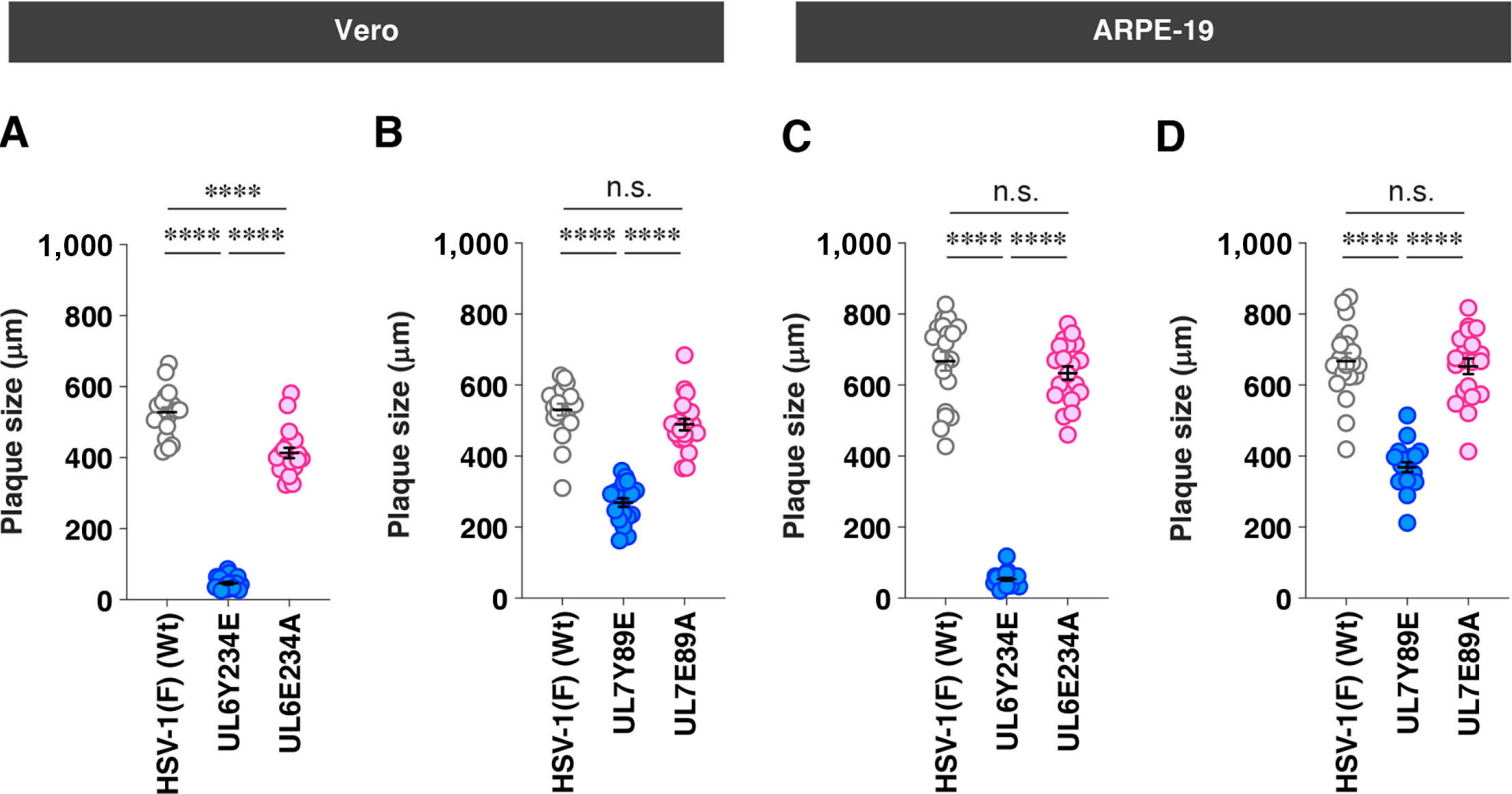
Effects of alanine substitution at UL6 and UL7 phosphorylation sites on plaque size in Vero and ARPE-19 cells. (**A–D**) Vero (**A and B**) and ARPE-19 (**C and D**) cells were infected with wild-type HSV-1(F) (**A–D**), YK443 (UL6 Y234E) (**A and C**), YK445 (UL6 E234A) (**A and C**), YK482 (UL7 Y89E) (**B and D**), and YK489 (UL7 E89A) (**B and D**) under plaque assay conditions. The diameters of 20 single plaques for each of the indicated viruses were measured 2 days post-infection. Each point represents a single plaque size. The horizontal line and error bars indicate the mean ± SEM for each group. Statistical significance was assessed by one-way ANOVA followed by Tukey’s test. ****, *P* < 0.0001 and n.s., not significant.

AlphaFold3 structural modeling predicted that the phenylalanine and glutamic acid substitutions at UL6 Tyr-234 and UL7 Tyr-89 would not disrupt the secondary structures of these HSV-1 proteins (Fig. 6). To further evaluate structural similarity, template modeling (TM) scores (scores ≥ 0.5 suggest minimal changes to the overall protein folding) (29) were calculated by comparing each mutant with its corresponding wild-type protein. The TM scores for UL6Y234F or UL6Y234E versus wild-type UL6 were both 0.84, and those for UL7Y89F or UL7Y89E versus wild-type UL7 were both 0.91 (Fig. 6). These results suggest that the amino acid substitutions at UL6 Tyr-234 and UL7 Tyr-89 have minimal effects on both the secondary structures and overall conformations of these HSV-1 proteins. However, substitution of tyrosine with glutamic acid introduces a negatively charged residue while eliminating the aromatic and hydrophobic properties of the tyrosine side chain, raising the possibility that local conformational changes unrelated to phospho-mimicry may contribute to the observed phenotypes. To address this possibility, we generated additional recombinant viruses [YK445 (UL6E234A) and YK489 (UL7E89A)] (Fig. 2), in which the glutamic acid substitutions at UL6 Tyr-234 and UL7 Tyr-89 were further replaced with alanine (UL6E234A and UL7E89A). Alanine substitution removes the bulky aromatic side chain and reduces hydrophobicity while avoiding the introduction of a negatively charged residue, allowing assessment of potential local structural effects independent of phosphomimetic substitution. In both Vero and ARPE-19 cells, progeny virus yields of UL6E234A and UL7E89A were significantly higher than those of UL6Y234E and UL7Y89E, respectively, at both MOIs of 3 and 0.01 (Fig. 7). Consistent with these results, YK445 (UL6E234A) and YK489 (UL7E89A) formed plaques that were significantly larger than those produced by YK441 (UL6Y234E) and YK482 (UL7Y89E) in both cell types (Fig. 8).

Collectively, these results indicate that phosphomimetic mutations at UL6 Tyr-234 and UL7 Tyr-89, but not at UL6 Tyr-231, reduce HSV-1 replication and cell-cell spread in Vero and ARPE-19 cells. This suggests that phosphorylation at UL6 Tyr-234 and UL7 Tyr-89, but not at UL6 Tyr-231, can downregulate the functions of these HSV-1 proteins in cultured cells. Thus, the results are in agreement with our prediction described above that highly conserved phosphorylation sites across the genus *Simplexvirus* are functionally relevant. Notably, the phenotypes of YK445 (UL6E234A) and YK489 (UL7E89A) were indistinguishable from those of wild-type HSV-1(F), except that progeny virus yields at an MOI of 3 and plaque sizes of UL6E234A in Vero cells were slightly but significantly lower than those of wild-type HSV-1(F) (Fig. 7 and 8). These results leave open the possibility that the glutamic acid substitution at UL6 Tyr-234 may have effects other than those related to phosphorylation. Given these considerations regarding UL6 Tyr-234, together with our previous reports on the importance of UL7 itself and/or its serine phosphorylation in HSV replication and pathogenicity (30, 31), we prioritized further investigation into the functional significance of UL7 Tyr-89 phosphorylation both *in vitro* and *in vivo*.

### Effects of phosphorylation at UL7 Tyr-89 on HSV-1 infection in cultured cells

HSV UL7 is a structural component of the virion tegument, a proteinaceous layer located between the nucleocapsid and the envelope (21, 32), and it plays important roles in HSV-1 replication and cell-to-cell spread in cultured cells (21, 22, 31), as well as in viral replication and pathogenicity *in vivo* (33). It has also been suggested, though not conclusively demonstrated, that UL7 is involved in final (cytoplasmic) envelopment in conjunction with another tegument protein, UL51 (21). To investigate the effects of phosphorylation at UL7 Tyr-89 on HSV-1 infection in cultured cells in more detail, we examined the effects of non-phosphorylatable and phosphomimetic mutations at UL7 Tyr-89, as well as the UL7 null mutation, on viral protein accumulation, subcellular localization of UL7, virion morphogenesis, viral growth kinetics, and viral cell-to-cell spread, by analyzing Vero cells infected with wild-type HSV-1(F), YK480 (UL7Y89F), YK481 (UL7Y89F-repair), YK482 (UL7Y89E), YK483 (UL7Y89E-repair), YK484 (ΔUL7), or YK485 (ΔUL7-repair). The results were as follows.

**Fig 9:**
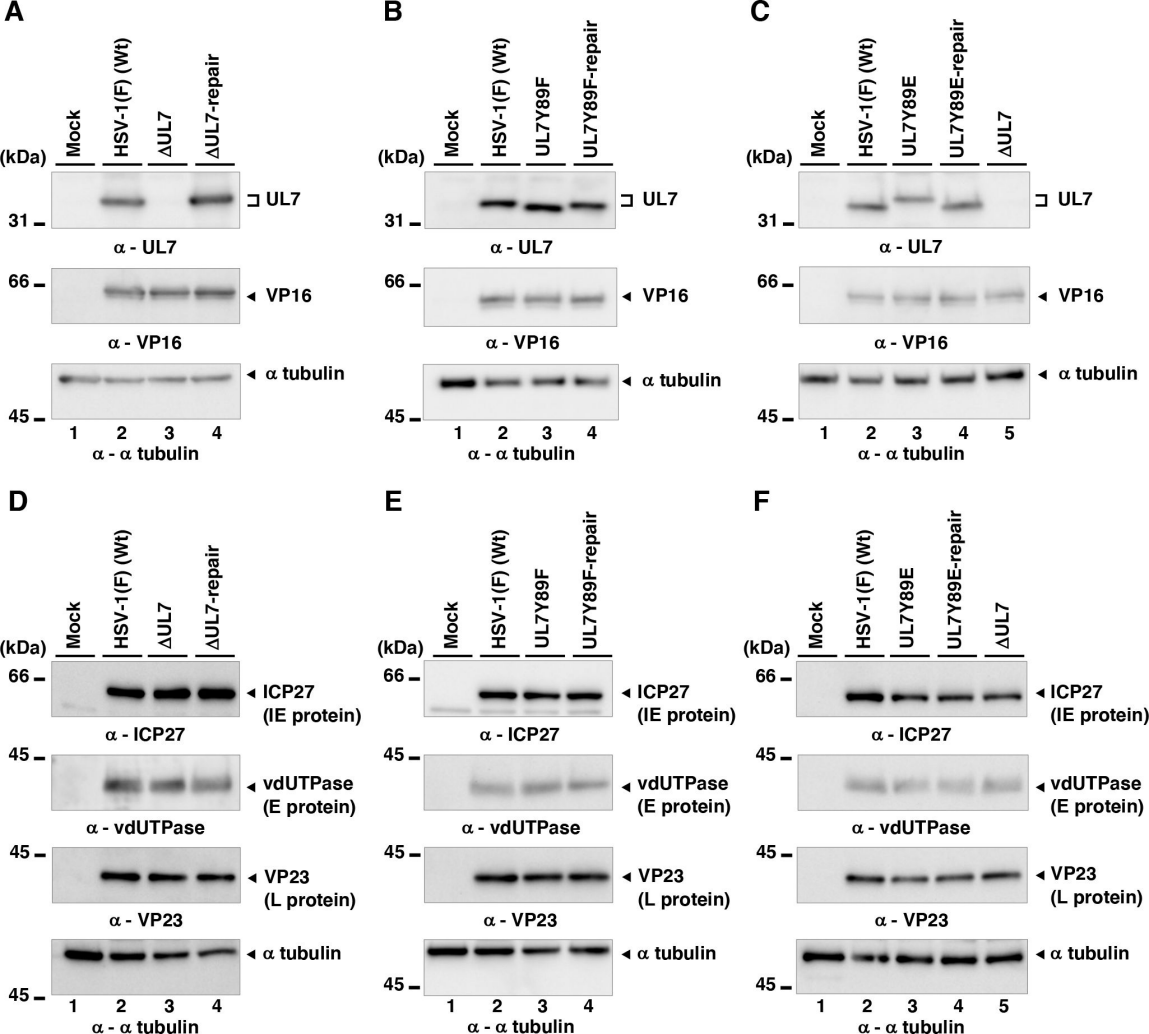
Effects of the mutations in UL7 on the expression of viral proteins in infected cells. (A–F) Vero cells were infected with wild-type HSV-1(F) (A–F), YK484 (ΔUL7) (A, C, D, and F), YK485 (ΔUL7-repair) (A and D), YK480 (UL7 Y89F) (**B and E**), YK481 (UL7 Y89F-repair) (**B and E**), YK482 (UL7 Y89E) (**C and F**), and YK483 (UL7 Y89E-repair) (**C and F**) at an MOI of 3, harvested 18 h post-infection, and analyzed by immunoblotting with the indicated antibodies. Digital images are representative of three independent experiments. A molecular mass marker is indicated on the left.

**Fig 10:**
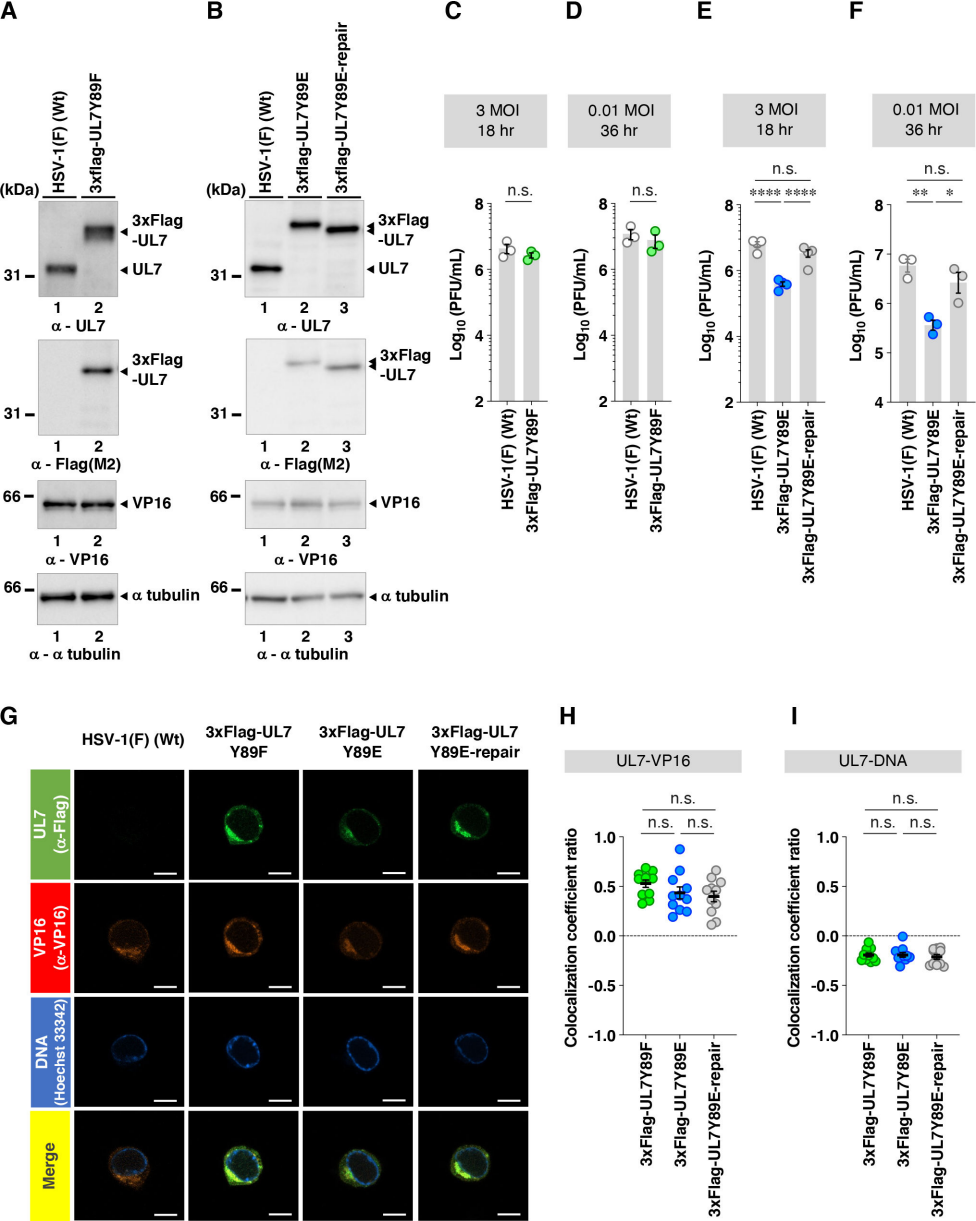
Effects of the mutations in UL7 on its localization and co-localization with VP16 in infected cells. (**A and B**) Vero cells were infected with wild-type HSV-1(F) (**A and B**), YK486 (3xFlag-UL7Y89F) (**A**), YK487 (3xFlag-UL7Y89E) (**B**), and YK488 (3xFlag-UL7Y89E-repair) (**B**) at an MOI of 3, harvested 18 h post-infection, and analyzed by immunoblotting with the indicated antibodies and Hoechst 33342. Digital images are representative of three independent experiments. A molecular mass marker is indicated on the left. (C–F) Vero cells were infected with wild-type HSV-1(F) (C–F), YK486 (3xFlag-UL7Y89F) (**C and D**), YK487 (3xFlag-UL7Y89E) (**E and F**), and YK488 (3xFlag-UL7Y89E-repair) (**E and F**) at MOIs of 3 (**C and E**) or 0.01 (**D and F**). Total virus from cell culture supernatants and infected cells was harvested 18 h (**C and E**) or 36 h (**D and F**) post-infection and assayed on Vero cells. Each data point represents the mean ± SEM of the results of three independent experiments. (**G**) Vero cells were infected with wild-type HSV-1(F), YK486 (3xFlag-UL7Y89F), YK487 (3xFlag-UL7Y89E), and YK488 (3xFlag-UL7Y89E-repair) at an MOI of 3, fixed at 18 h post-infection, permeabilized, stained with the indicated antibodies, and examined by confocal microscopy. White scale bar, 10 mm. (**H and I**) Colocalization between 3xFlag-tagged UL7 and VP16 (**H**), and between 3xFlag-tagged UL7 and DNA (**I**), was quantified using Pearson’s colocalization coefficient. Each data point represents one cell (*n* = 11 cells), collected across at least three independent experiments. Data are shown as the mean ± SEM indicated. Statistical significance was assessed by an unpaired two-tailed Student’s *t*-test (C and D) and one-way ANOVA followed by Tukey’s test (E, F, H, and I). *, *P* < 0.05; **, *P* < 0.01; ****, *P* < 0.0001; and n.s., not significant.

**Fig 11:**
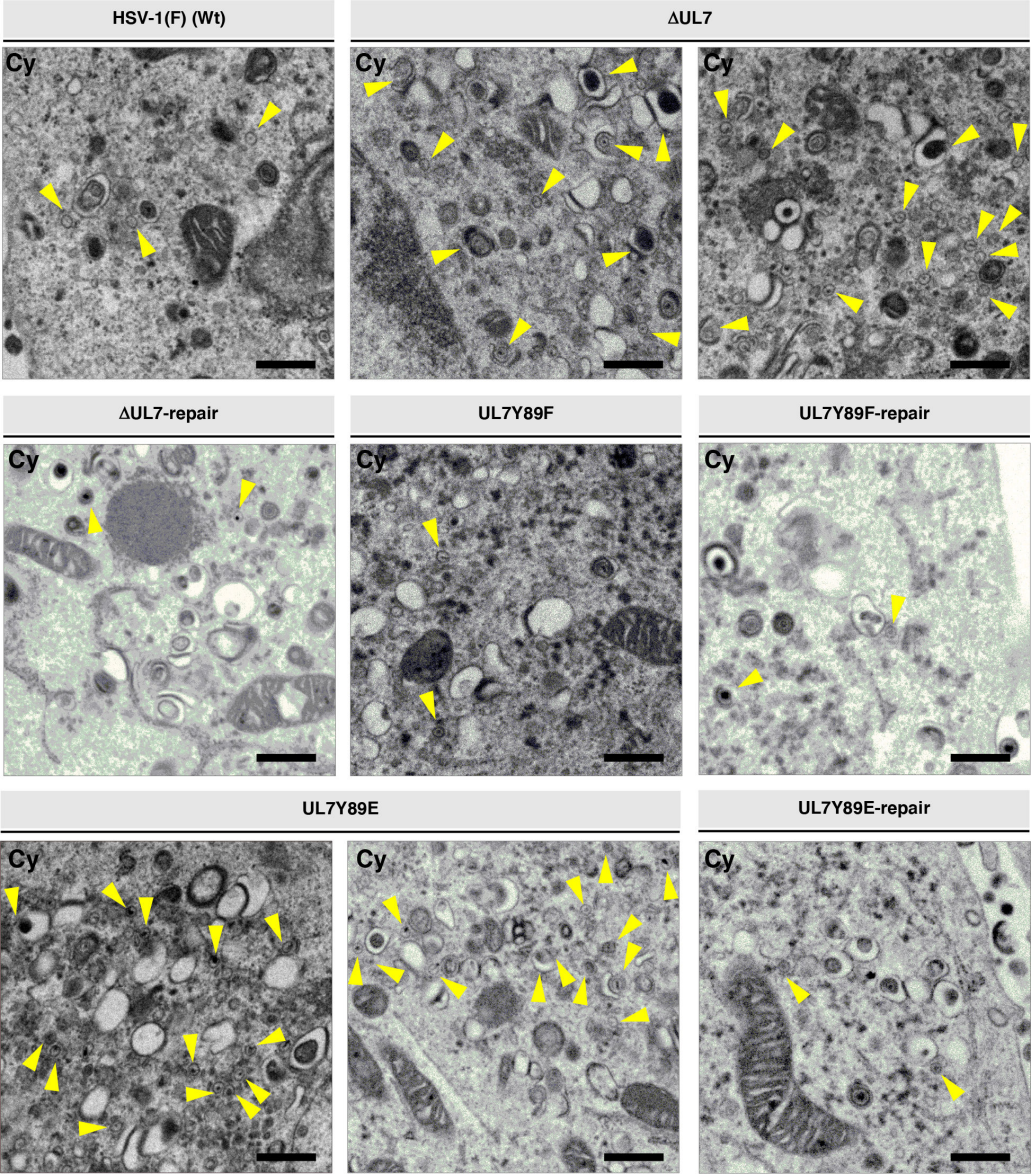
Ultrastructural analysis of the effects of mutations in UL7 on viral final envelopment in the cytoplasm of infected cells. Vero cells were infected with wild-type HSV-1(F), YK484 (ΔUL7), YK485 (ΔUL7-repair), YK480 (UL7 Y89F), YK481 (UL7 Y89F-repair), YK482 (UL7 Y89E), and YK483 (UL7 Y89E-repair) at an MOI of 3, fixed at 18 h post-infection, embedded, sectioned, stained, and examined by transmission electron microscopy. Digital images are representative of three independent experiments. Black scale bar, 500 nm. Yellow arrows indicate unenveloped capsids and partially enveloped capsids.

**Fig 12:**
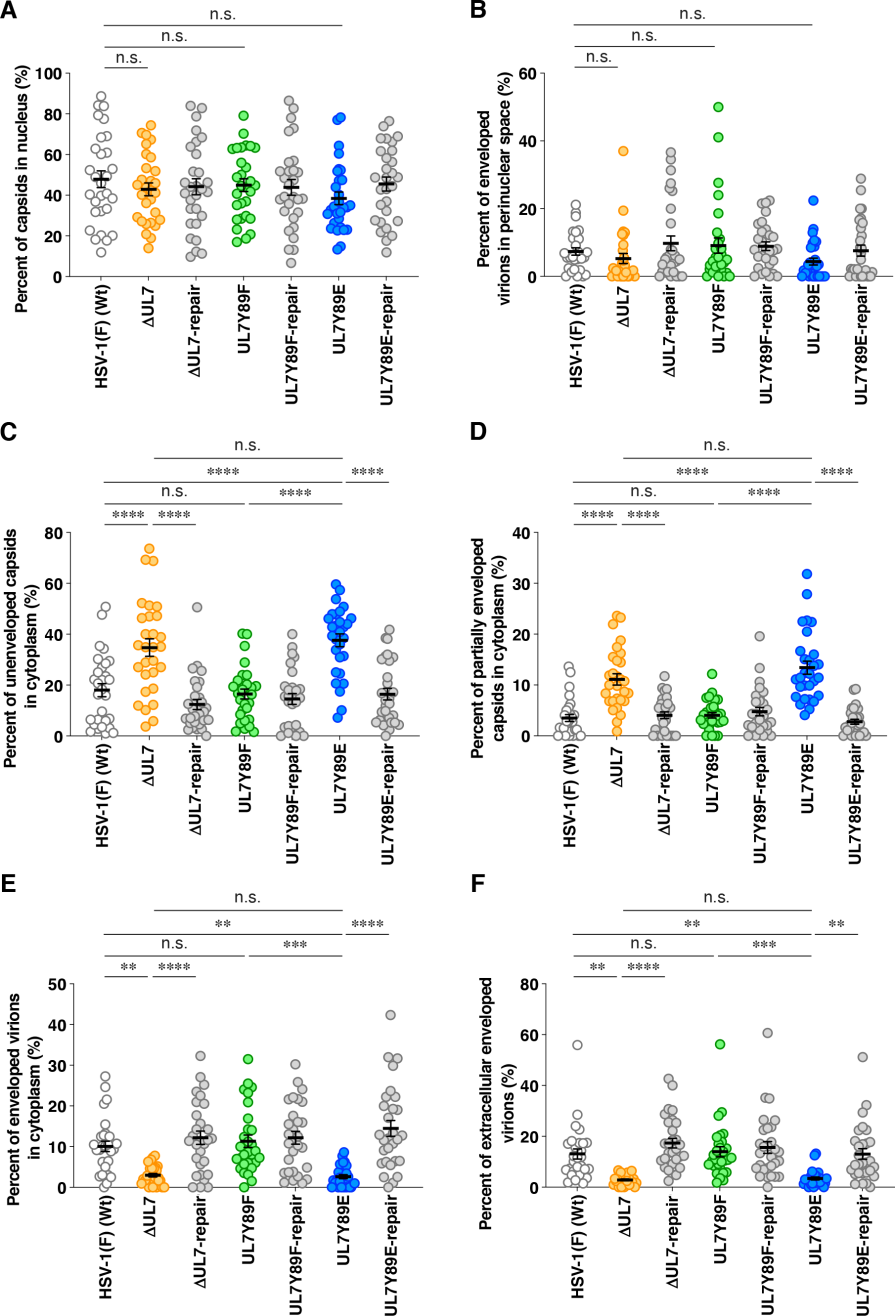
Effects of the mutations in UL7 on the distribution of viral particles in HSV-1-infected cells. Quantitative analysis of viral morphogenesis was performed on electron micrographs obtained in Fig. 8. Data represent the mean ± SEM of the percentages of viral particles corresponding to each morphogenetic step—capsids in the nucleus (**A**), enveloped virions in the perinuclear space (**B**), unenveloped capsids in the cytoplasm (**C**), partially enveloped capsids in the cytoplasm (**D**), enveloped virions in the cytoplasm (**E**), and extracellular enveloped virions (**F**)—from at least three independent experiments (*n* = 29 cells in total). Statistical significance was assessed by one‐way ANOVA followed by Tukey’s test. **, *P* < 0.01; ***, *P* < 0.001; ****, *P* < 0.0001; and n.s., not significant.

**Fig 13:**
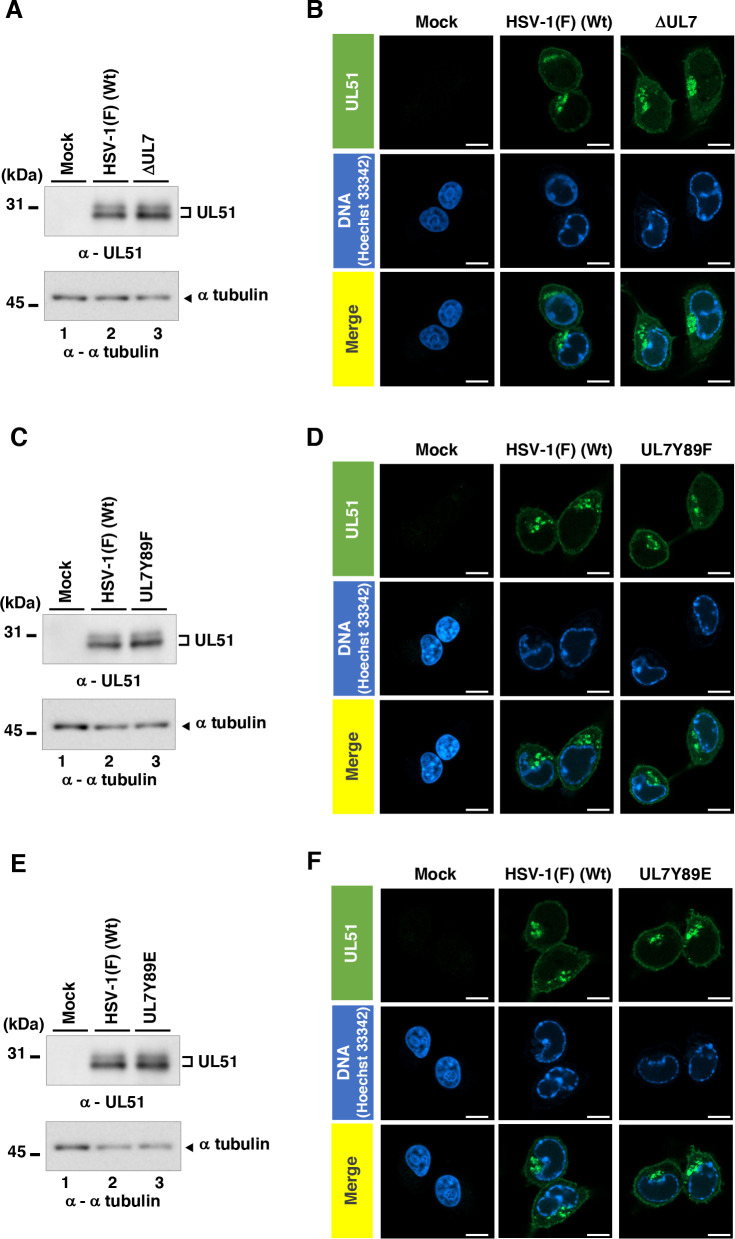
Effects of the mutations in UL7 on the accumulation and localization of its binding partner UL51 in infected cells. (**A, C, and E**) Vero cells were infected with wild-type HSV-1(F) (**A, C, and E**), YK484 (ΔUL7) (**A**), YK480 (UL7 Y89F) (**C**), and YK482 (UL7 Y89E) (**E**) at an MOI of 3, harvested 18 h post-infection, and analyzed by immunoblotting with the indicated antibodies. Digital images are representative of three independent experiments. A molecular mass marker is indicated on the left. (**B, D, and F**) Vero cells were infected with wild-type HSV-1(F) (B, D, and F), YK484 (ΔUL7) (**B**), YK480 (UL7 Y89F) (**D**), and YK482 (UL7 Y89E) (**F**) at an MOI of 3, fixed at 18 h post-infection, permeabilized, stained with the indicated antibody and Hoechst 33342, and examined by confocal microscopy. Digital images are representative of three independent experiments. White scale bar, 10 µm.

**Fig 14:**
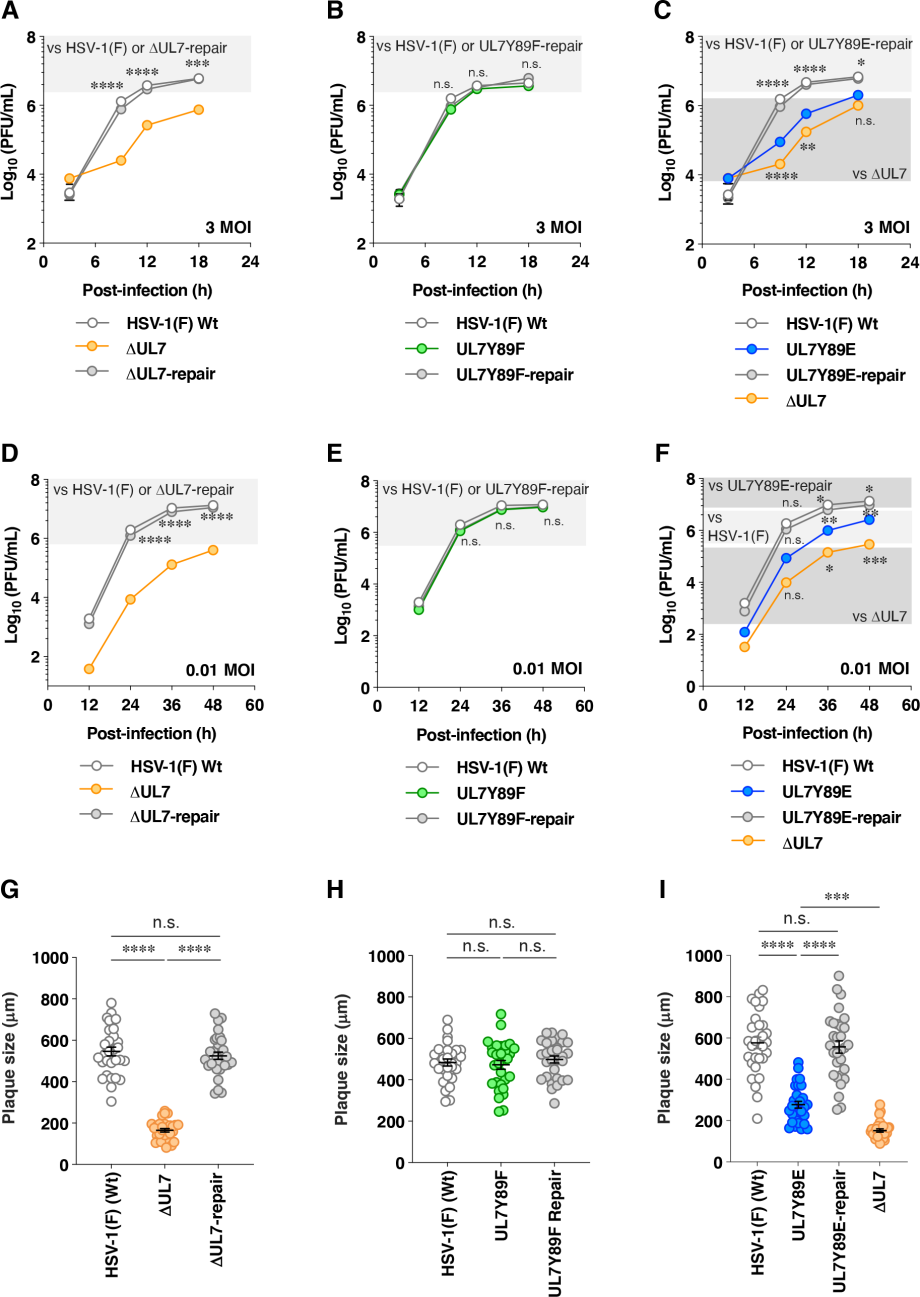
Effects of mutations in UL7 on viral growth kinetics and plaque size in cell cultures. (A–F) Vero cells were infected with wild-type HSV-1(F) (A–F), YK484 (ΔUL7) (A, B, E, and F), YK485 (ΔUL7-repair) (A, B, E, and F), YK480 (UL7 Y89F) (**C and D**), YK481 (UL7 Y89F-repair) (**C and D**), YK482 (UL7 Y89E) (**E and F**), and YK483 (UL7 Y89E-repair) (**E and F**) at an MOI of 3 (A, C, and E) or 0.01 (B, D, and F). Total virus from the cell culture supernatants and infected cells was harvested at the indicated times and assayed on Vero cells. Each data point represents the mean ± SEM of the results of four (A–E) and five (**F**) independent experiments. Statistical significance was assessed by one-way ANOVA followed by Tukey’s test. The *P*-values for comparisons between YK484 (ΔUL7) (**A and D**), YK480 (UL7 Y89F) (**B and E**), YK482 (UL7 Y89E) (**C and F**), and wild-type HSV-1(F) together with their corresponding repair viruses are indicated. In panels **C and F**, the *P*-values for comparisons between YK484 (ΔUL7) and YK482 (UL7 Y89E) are also shown. *, *P* < 0.05; ***, *P* < 0.001; ****, *P* < 0.0001; and n.s., not significant. (G–I) Vero cells were infected with wild-type HSV-1(F) (G–I), YK484 (ΔUL7) (**G and I**), YK485 (ΔUL7-repair) (**G**), YK480 (UL7 Y89F) (**H**), YK481 (UL7 Y89F-repair) (**H**), YK482 (UL7 Y89E) (**I**), and YK483 (UL7 Y89E-repair) (**I**) under plaque assay conditions. The diameters of 30 single plaques for each of the indicated viruses were measured 2 days post-infection. Each point represents a single plaque size. The horizontal line and error bars indicate the mean ± SEM for each group. Statistical significance was assessed by one-way ANOVA followed by Tukey’s test. ***, *P* < 0.001; ****, *P* < 0.0001; and n.s., not significant.

- As expected, UL7 was detected by immunoblotting in lysates of cells infected with wild-type HSV-1(F) or YK485 (ΔUL7-repair) but was absent in those infected with YK484 (ΔUL7), whereas VP16 levels in YK484 (ΔUL7)-infected cells were comparable to those in cells infected with wild-type HSV-1(F) or YK485 (ΔUL7-repair) (Fig. 9A). Cells infected with YK480 (UL7Y89F) or YK482 (UL7Y89E) accumulated UL7 at levels comparable to those in cells infected with wild-type HSV-1(F) or each of their repaired viruses, YK481 (UL7Y89F-repair) and YK483 (UL7Y89E-repair), respectively (Fig. 9B and C). HSV-1 proteins are categorized into three major classes—immediate-early (IE), early (E), and late (L)—whose expressions are regulated coordinately and ordered sequentially in a cascade fashion (14). Cells infected with YK484 (ΔUL7), YK480 (UL7Y89F), or YK482 (UL7Y89E) accumulated representative IE (ICP27, encoded by UL54 gene), E (viral dUTPase, encoded by UL50 gene), and L (VP23, encoded by UL38 gene) proteins at levels comparable to those in cells infected with wild-type HSV-1(F) or each of their repaired viruses, YK485 (ΔUL7-repair), YK481 (UL7Y89F-repair), and YK483 (UL7Y89E-repair), respectively (Fig. 9D through F). These results indicate that mutations at UL7 Tyr-89 have little effect on the accumulation of UL7 itself or of other representative IE, E, and L proteins in HSV-1-infected Vero cells.
- Unfortunately, neither the polyclonal anti-UL7 antibody available in our laboratory (30) nor the previously reported monoclonal anti-UL7 antibody (22) reliably detected UL7 in HSV-1-infected cells by immunofluorescence microscopy. Therefore, we constructed YK486 (3xFlag-UL7Y89F) and YK487 (3xFlag-UL7Y89E), in which a 3xFlag tag sequence was inserted into the putative loop region of UL7 (Fig. 2 and 6D), and Tyr-89 was substituted with phenylalanine or glutamic acid, respectively (Fig. 2). Additionally, we constructed a repaired virus, YK488 (3xFlag-UL7Y89E-repair), in which the Y89E mutation in YK487 (3xFlag-UL7Y89E) was restored, to serve as a control (Fig. 2). As expected, 3xFlag-tagged UL7Y89F, UL7Y89E, and wild-type UL7 were detected by immunoblotting with anti-Flag and/or anti-UL7 antibodies in lysates of Vero cells infected with YK486 (3xFlag-UL7Y89F), YK487 (3xFlag-UL7Y89E), or YK488 (3xFlag-UL7Y89E-repair), respectively (Fig. 10A and B). Progeny virus yields in Vero cells infected with YK486 (3xFlag-UL7Y89F) or YK488 (3xFlag-UL7Y89E-repair) at an MOI of 3 or 0.01 for 18 or 36 h, respectively, were comparable to those in cells infected with wild-type HSV-1(F) (Fig. 10C and D). In contrast, progeny virus yields in cells infected with YK487 (3xFlag-UL7Y89E) under the same conditions were significantly lower than those in cells infected with wild-type HSV-1(F) or YK488 (3xFlag-UL7Y89E-repair) (Fig. 10E and F). These results are in agreement with those observed for the untagged recombinant viruses, YK480 (UL7Y89F), YK482 (UL7Y89E), and YK483 (UL7Y89E-repair) (Fig. 3F and G), indicating that tagging UL7 with the 3xFlag epitope has minimal effect on HSV-1 replication in Vero cells. As shown in Fig. 7G, 3xFlag-tagged UL7Y89F, UL7Y89E, and wild-type UL7 were detected by immunofluorescence microscopy using the anti-Flag antibody and were similarly distributed throughout the cytoplasm, with predominant localization in the juxtanuclear region of Vero cells infected with YK486 (3xFlag-UL7Y89F), YK487 (3xFlag-UL7Y89E), or YK488 (3xFlag-UL7Y89E-repair), respectively (Fig. 10G). In these infected cells, VP16, another tegument protein, exhibited a distribution comparable to that of wild-type and mutant 3xFlag-UL7 and co-localized with each form (Fig. 10G). Pearson’s correlation analysis (1 = perfect colocalization; 0 = no correlation; and −1 = complete anti-correlation) yielded coefficients ranging from 0.4 to 0.53 for VP16 and each of the 3xFlag-UL7 forms (Fig. 10H), in contrast to values between −0.19 and −0.21 for DNA and each of the 3xFlag-UL7 forms (Fig. 10I), confirming the efficient co-localization of UL7 with VP16. These results indicate that mutations at UL7 Tyr-89 are not required for the proper localization of UL7 and VP16 in HSV-1-infected cells.
- To investigate the effects of UL7 Tyr-89 phosphorylation on virion morphogenesis, we quantified the number of virus particles at different morphogenetic stages by electron microscopy. As shown in Fig. 11, partially enveloped capsids were readily detectable in the cytoplasm of Vero cells infected with YK484 (ΔUL7) or YK482 (UL7Y89E), with both unenveloped and partially enveloped capsids aberrantly accumulating in the cytoplasm of these infected cells. In Vero cells infected with YK484 (ΔUL7) or YK482 (UL7Y89E), 11% and 13% of virus particles, respectively, were partially enveloped capsids in the cytoplasm (Fig. 12; Table 1). However, only 2.8% to 4.1% of virus particles were partially enveloped capsids in the cytoplasm of cells infected with wild-type HSV-1(F) or each of the respective repaired viruses [YK485 (ΔUL7-repair) and YK483 (UL7Y89E-repair)], representing a significant 2.7- to 4.8-fold reduction compared to those infected with YK484 (ΔUL7) or YK482 (UL7Y89E) (Fig. 12; Table 1). Similarly, 46% and 51% of virus particles in cells infected with YK484 (ΔUL7) or YK482 (UL7Y89E), respectively, were either unenveloped or partially enveloped capsids in the cytoplasm. In contrast, in cells infected with wild-type HSV-1(F) or each of the respective repaired viruses [YK485 (ΔUL7-repair) and YK483 (UL7Y89E-repair)], 17%–22% of virus particles were such capsids, representing a significant 2.1- to 3.1-fold reduction compared to those in cells infected with YK484 (ΔUL7) or YK482 (UL7Y89E) (Fig. 12; Table 1). Conversely, in cells infected with YK484 (ΔUL7) or YK482 (UL7Y89E), 5.9% and 6.1% of virus particles, respectively, were enveloped virions in the cytoplasm and the extracellular space, whereas in cells infected with wild-type HSV-1(F) or each of the respective repaired viruses [YK485 (ΔUL7-repair) and YK483 (UL7Y89E-repair)], this proportion increased to between 23% and 30%, representing a significant 3.9- to 5.0-fold increase compared to those in cells infected with YK484 (ΔUL7) or YK482 (UL7Y89E) (Fig. 12; Table 1). The proportion of total virus particles in the nucleus and the perinuclear space was comparable across cells infected with wild-type HSV-1(F) or each of the recombinant viruses (43%–55%) (Fig. 12; Table 1). We verified that the UL7 null, the UL7-Y89E, and the UL7-Y89F mutations had little effect on the accumulation and localization of UL51 (Fig. 13), which interacts with UL7 and functions cooperatively with it (21, 22). Thus, both the UL7 null and the UL7-Y89E phosphomimetic mutations induced comparable levels of aberrant accumulation of unenveloped and partially enveloped capsids, along with a marked reduction in fully enveloped virions in the cytoplasm and the extracellular space. These results indicate that UL7 is required for efficient final envelopment without detectable effects on UL51 accumulation or subcellular localization and that phosphomimetic mutation at UL7 Tyr-89 significantly impairs this virion morphogenetic step.
- In agreement with previous reports (22, 31, 33), YK484 (ΔUL7) replicated significantly less efficiently in Vero cells than wild-type HSV-1(F) and YK485 (ΔUL7-repair) at both MOIs of 3 and 0.01 (Fig. 14A and D). Similarly, YK482 (UL7Y89E) replicated significantly less efficiently than HSV-1(F) and YK483 (UL7Y89E-repair) at both MOIs (Fig. 14C and F). However, the reduction in viral replication of YK482 (UL7Y89E) was less pronounced than that of YK484 (ΔUL7), and progeny virus yields of YK484 (ΔUL7) were significantly lower than those of YK482 (UL7Y89E) at 9 and 12 h at an MOI of 3, and 36 and 48 h post infection at an MOI of 0.01 (Fig. 14C and F). In contrast, the growth kinetics of YK480 (UL7Y89F) at both MOIs were almost identical to those of HSV-1(F) and YK483 (UL7Y89E-repair) (Fig. 14B and E). In agreement with the observed growth kinetics, YK484 (ΔUL7) produced markedly smaller plaques than wild-type HSV-1(F) and YK485 (ΔUL7-repair), whereas YK480 (UL7Y89F) formed plaques comparable in size to those of wild-type HSV-1(F) (Fig. 14G and H). YK482 (UL7Y89E) produced plaques that were smaller than those of HSV-1(F) and YK483 (UL7Y89E-repair), but larger than those of YK484 (ΔUL7) (Fig. 14I). These results indicate that UL7 null and phosphomimetic mutations at UL7 Tyr-89 significantly reduce HSV-1 replication and plaque size in cultured cells, although the magnitude of the effect differs between the two mutants.

**TABLE 1:** Effects of the mutations in UL7 on the distribution of viral particles in HSV-1-infected Vero cells

Virus	Capsids in the nucleus	% of virus particles in the morphogenetic stage ± SEM (no. of particles in the stage)					Total counted (particles/cells)
		Enveloped virions in the perinuclear space	Unenveloped capsids in the cytoplasm	Partially enveloped capsids in the cytoplasm	Enveloped virions in the cytoplasm	Extracellular enveloped virions	
HSV-1 (F) (Wt)	47.9 ± 4.1 (1,426)	7.4 ± 1.1 (198)	18.0 ± 2.6 (510)	3.5 ± 0.7 (104)	10.0 ± 1.3 (281)	13.1 ± 2.0 (349)	2,868/29
ΔUL7	43.0 ± 3.1 (1,132)	5.3 ± 1.5 (155)	34.8 ± 3.4 (907)	11.1 ± 1.1 (268)	3.0 ± 0.4 (77)	2.9 ± 0.4 (74)	2,615/29
ΔUL7-repair	44.2 ± 4.0 (1,197)	9.8 ± 2.2 (228)	12.4 ± 2.0 (278)	4.1 ± 0.7 (91)	12.2 ± 1.6 (277)	17.3 ± 1.9 (361)	2,432/29
UL7Y89F	45.0 ± 3.1 (1,136)	9.1 ± 2.3 (171)	16.5 ± 2.0 (363)	4.0 ± 0.5 (96)	11.4 ± 1.5 (248)	14.0 ± 2.0 (300)	2,314/29
UL7Y89F-repair	43.9 ± 3.9 (1,204)	8.9 ± 1.3 (189)	14.6 ± 2.1 (321)	4.8 ± 0.8 (102)	12.2 ± 1.6 (281)	15.6 ± 2.4 (343)	2,440/29
UL7Y89E	38.5 ± 3.1 (798)	4.4 ± 1.0 (88)	37.6 ± 2.5 (773)	13.4 ± 1.3 (246)	2.6 ± 0.5 (52)	3.4 ± 0.6 (65)	2,022/29
UL7Y89E-repair	45.6 ± 3.4 (1,095)	7.6 ± 1.5 (161)	16.5 ± 2.3 (341)	2.8 ± 0.5 (60)	14.5 ± 1.9 (294)	13.1 ± 2.1 (267)	2,218/29

### Effects of phosphorylation at UL7 Tyr-89 on HSV-1 replication and pathogenicity in the CNS of mice

**Fig 15:**
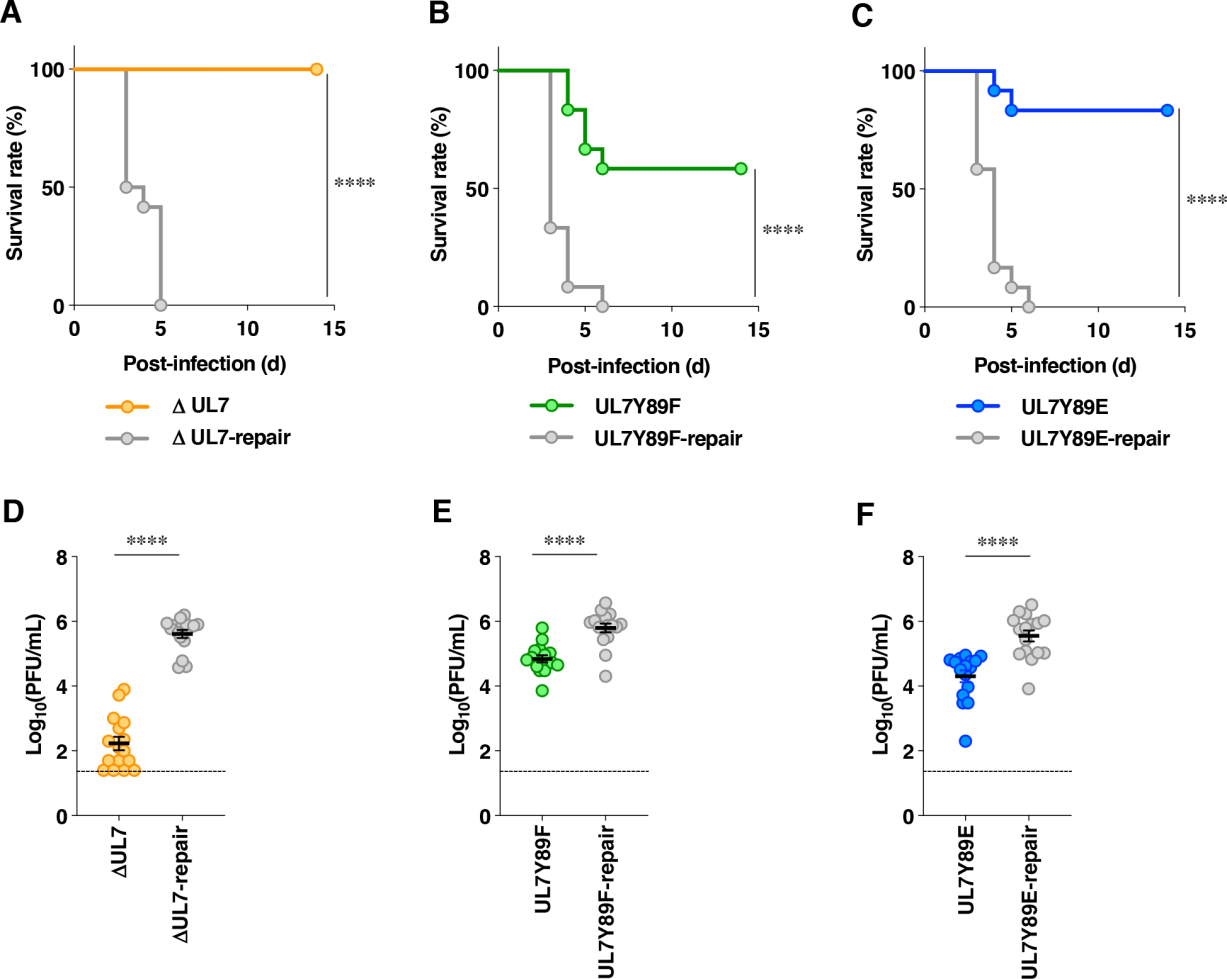
Effects of the mutations in UL7 on the mortality rate and viral replication in the brains of mice following intracranial infection. (**A–C**) Three-week-old female mice (*n* = 12 per group) were infected intracranially with 1 × 10^3^ PFU/head of YK484 (ΔUL7) (**A**), YK485 (ΔUL7-repair) (**A**), YK480 (UL7 Y89F) (**B**), YK481 (UL7 Y89F-repair) (**B**), YK482 (UL7 Y89E) (**C**), and YK483 (UL7 Y89E-repair) (**C**) and were monitored for 14 days. Differences in the mortality rate of infected mice were analyzed statistically by the log-rank test. ****, *P* < 0.0001. (**D–F**) Three-week-old female mice (*n* = 14 per group) were infected intracranially as described in panels **A–C**, and viral titers in the brains of mice were assayed on Vero cells. Each data point represents the virus titer from one mouse, with the horizontal line and error bars indicating the mean ± SEM. Statistical significance was assessed by an unpaired two-tailed Student’s *t*-test. ****, *P* < 0.0001 and n.s., not significant.

The phosphomimetic mutation at UL7 Tyr-89 impaired HSV-1 replication, cell-to-cell spread, and final envelopment in Vero cells, exhibiting phenotypes similar to those of the UL7 deletion mutation (Fig. 3, 5, 7, 8, 10, 11, 12 and 14). In contrast, the non-phosphorylatable mutation at this site had little effect on HSV-1 replication, cell-to-cell spread, and final envelopment (Fig. 3, 5, 7, 8, 10, 11, 12 and 14). These results raised the possibility that UL7 Tyr-89 is either not phosphorylated or is phosphorylated only at a level insufficient to affect HSV-1 replication, cell-to-cell spread, or final envelopment in Vero cells. To elucidate the physiological relevance of Tyr-89 phosphorylation in UL7, we next examined the effects of non-phosphorylatable and phosphomimetic mutations at UL7 Tyr-89, as well as the UL7 null mutation, on HSV-1 infection *in vivo*. To this end, 3-week-old female ICR mice were infected intracranially with 1 × 10^3^ PFU/head of YK484 (ΔUL7), YK485 (ΔUL7-repair), YK480 (UL7 Y89F), YK481 (UL7 Y89F-repair), YK482 (UL7 Y89E), or YK483 (UL7 Y89E-repair). The survival of the infected mice was monitored for 14 days, and virus titers in infected mouse brains were assayed at 3 days post-infection. As shown in Fig. 15A through C, the survival curves of mice infected with each of the repaired viruses [YK485 (ΔUL7-repair), YK481 (UL7 Y89F-repair), and YK483 (UL7 Y89E-repair)] were similar, with all infected mice succumbing to infection by 5–6 days post-infection. In contrast, the survival rates of mice infected with YK484 (ΔUL7), YK480 (UL7 Y89F), or YK482 (UL7 Y89E) were significantly higher than those of mice infected with each of their respective repaired viruses [YK485 (ΔUL7-repair), YK481 (UL7 Y89F-repair), and YK483 (UL7 Y89E-repair)] (Fig. 15A through C). The survival outcomes varied among the mutant viruses: those infected with YK484 (ΔUL7) exhibited the highest survival rate of 100%, followed by YK482 (UL7 Y89E) at 83.3%, while YK480 (UL7 Y89F) showed the lowest survival rate at 53% (Fig. 15A through C). In agreement with the effects of the mutations in UL7 on mouse survival, viral titers in the brains of mice infected with YK484 (ΔUL7), YK480 (UL7 Y89F), or YK482 (UL7 Y89E) were significantly lower than those in mice infected with each of their respective repaired viruses [YK485 (ΔUL7-repair), YK481 (UL7 Y89F-repair), and YK483 (UL7 Y89E-repair)] (Fig. 15D through F). The reductions in viral titers in the brains of mice also varied among the mutant viruses: YK484 (ΔUL7) showed the greatest decrease (approximately 610-fold), followed by YK482 (UL7Y89E) with a 20-fold decrease, and YK480 (UL7Y89F) with a 9.0-fold decrease, relative to each of their respective repaired viruses (Fig. 15D through F). These results with YK484 (ΔUL7), YK480 (UL7Y89F), YK482 (UL7Y89E), and their repaired viruses indicate that UL7 null and the mutations at UL7 Tyr-89 significantly reduced HSV-1 replication and pathogenicity in the CNS of mice following intracranial infection.

### Effects of phosphorylation at UL7 Tyr-89 on HSV-1 replication and pathogenic manifestations in the eyes of mice

**Fig 16:**
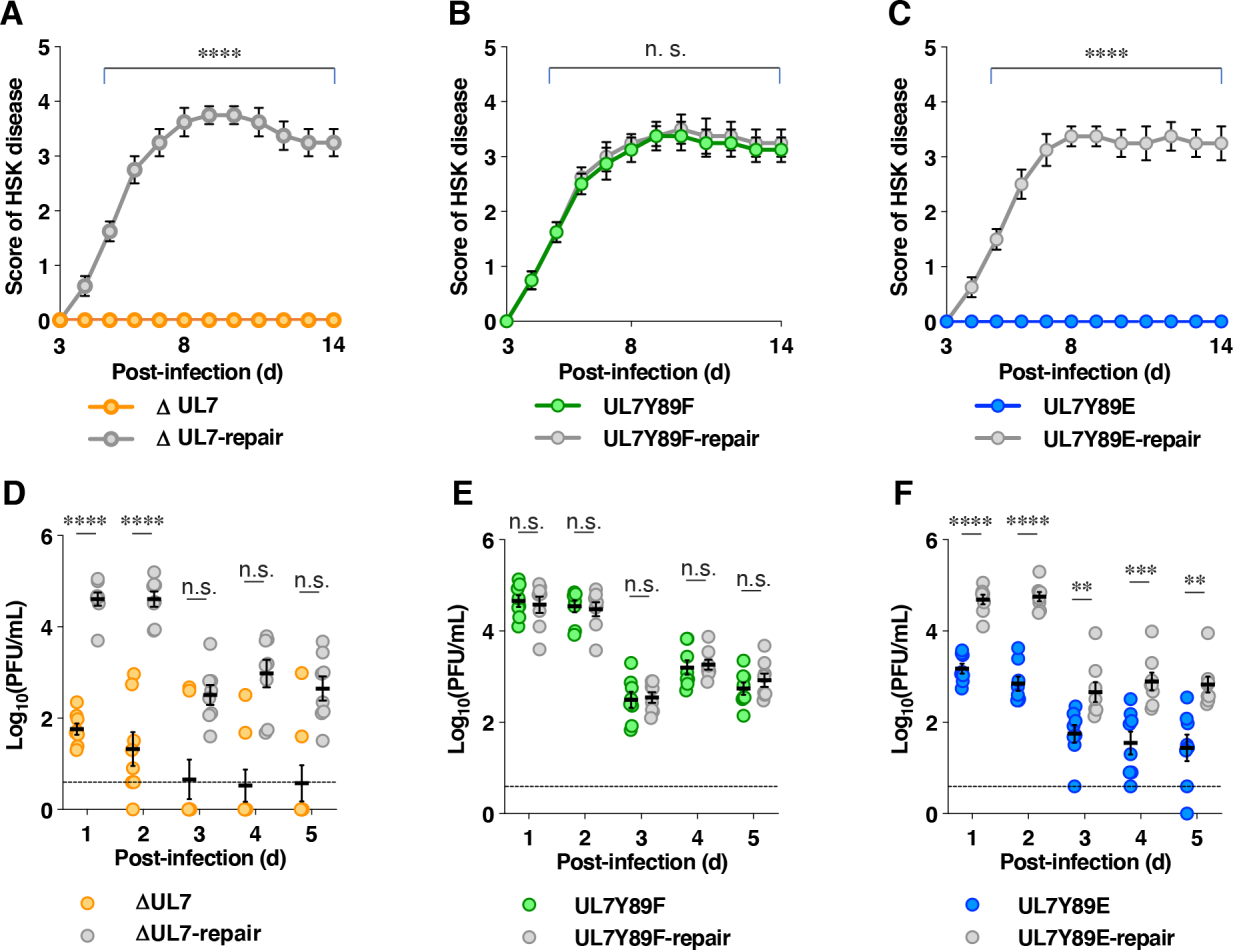
Effects of the mutations in UL7 on pathogenic effects in the eyes and viral replication in the tear film of mice following ocular infection. (**A–C**) Five-week-old female mice (*n* = 8 per group) were infected ocularly with 1 × 10^5^ PFU/eye of YK484 (ΔUL7) (**A**), YK485 (ΔUL7-repair) (**A**), YK480 (UL7 Y89F) (**B**), YK481 (UL7 Y89F-repair) (**B**), YK482 (UL7 Y89E) (**C**), and YK483 (UL7 Y89E-repair) (**C**) and scored for HSK every day for 14 days. Each data point represents the mean ± SEM of the scores. (**D–F**) The tear films of infected mice 1–5 days post-infection in the experiment described in panels **A–C** were harvested, and viral titers were assayed. Each data point represents the virus titer in the tear film of one mouse, with the horizontal line and error bars indicating the mean ± SEM for each group. Statistical significance was assessed by an unpaired two-tailed Student’s *t* test. The *P*-values for comparisons of data from 6 to 14 days post-infection in panels **A–C** are provided. **, *P* < 0.01; ***, *P* < 0.001; ****, *P* < 0.0001; and n.s., not significant. No mortality occurred, and data were obtained from all animals tested.

We also examined the effects of non-phosphorylatable and phosphomimetic mutations at UL7 Tyr-89, as well as the UL7 null mutation, on HSV-1 replication and pathogenic manifestations at a peripheral site of mice. To this end, 5-week-old female mice were infected ocularly with 1 × 10^5^ PFU/eye of YK484 (ΔUL7), YK485 (ΔUL7-repair), YK480 (UL7 Y89F), YK481 (UL7 Y89F-repair), YK482 (UL7 Y89E), or YK483 (UL7 Y89E-repair). The infected mice were observed daily for the development of herpes stromal keratitis (HSK) for 14 days, and virus titers detected in tear films were assayed at 1–5 days post-infection. In agreement with the results observed in mice intracranially infected with YK484 (ΔUL7) or YK482 (UL7 Y89E), mice ocularly infected with YK484 (ΔUL7) or YK482 (UL7 Y89E) exhibited significantly reduced severity of HSK compared to those infected with each of the respective repaired viruses [YK485 (ΔUL7-repair) and YK483 (UL7 Y89E-repair)] (Fig. 16A and C). Virus titers in the tear films of mice infected with YK484 (ΔUL7) or YK482 (UL7 Y89E) were also significantly lower than those of mice infected with YK485 (ΔUL7-repair) or YK483 (UL7 Y89E-repair), respectively (Fig. 16D and F). In contrast, although YK480 (UL7 Y89F) was significantly attenuated compared to its repaired virus YK481 (UL7 Y89F-repair) in mice following intracranial infection as described above (Fig. 15B and E), mice infected with YK480 (UL7 Y89F) exhibited similar severity of HSK to that observed in mice infected with YK481 (UL7 Y89F-repair) (Fig. 16B), and virus titers in their tear films were likewise comparable (Fig. 16E). These results indicate that UL7 is required for efficient viral replication and pathogenic manifestations in the eyes of mice following ocular infection. In this model, a phosphomimetic mutation at Tyr-89 was associated with reduced replication and disease, whereas the non-phosphorylatable mutation had minimal effect compared with the corresponding repaired virus, suggesting that a Tyr-89 phosphorylation does not appear to have a significant regulatory role under these conditions.

## DISCUSSION

By integrating genus-level conservation with viral phosphoproteomic data, we developed an evolution-guided framework to prioritize candidate phosphorylation sites with high potential for functional relevance from large-scale viral phosphoproteomes. Using this approach, we demonstrated that non-phosphorylatable and/or phosphomimetic mutations in UL6 Tyr-234 and UL7 Tyr-89, which are conserved in 100% and 88% of *Simplexvirus* species, respectively, resulted in significant phenotypic effects in cultured cells and/or mouse models of HSV-1 infection, suggesting that these sites are functionally relevant during HSV-1 infection. Thus, it appears that a conservation threshold of 88% or higher, which encompasses 46 phosphorylation sites, including UL6 Tyr-234 and UL7 Tyr-89, as shown in Fig. 1B and C, serves as a useful criterion for prioritizing candidate phosphorylation sites with high potential for functional relevance. Supporting this, several phosphorylation sites in HSV-1 proteins previously reported to be functionally important in viral infection also meet this conservation threshold. These include envelope glycoprotein B encoded by UL27 Tyr-889 (24), viral alkaline nuclease UL12 Tyr-371 (34), viral protein kinase UL13 Tyr-162 (35), viral uracil-DNA glycosylase encoded by UL2 Ser-302 (10), and viral transcriptional activator VP16 encoded by UL48 Ser-375 (36) (Fig. S1; Table S2; Table 2), even though only gB Tyr-889 was detected in the phosphoproteomic analysis by Kulej et al. (4) (Fig. S1; Table S2), while others were not (Table 2). Thus, further studies to investigate the virological significance of phosphorylation at the remaining 43 phosphorylation sites shown in Fig. S1 and Table S2, which meet this criterion, will be important to elucidate how phosphorylation contributes to the regulation and functional modulation of HSV-1 proteins and to evaluate the broader applicability of this approach. Such studies are currently underway in our laboratory. Moreover, additional investigation focusing on phosphorylation sites with lower conservation levels will be necessary to determine the minimal conservation threshold that allows accurate prioritization of functionally relevant candidate sites without compromising predictive performance.

**TABLE 2:** Conservation of functionally validated phosphorylation sites in HSV-1 or HSV-2 across the genus *Simplexvirus^a^*

Gene		UL2	UL7		UL12	UL13		UL21		UL27		UL35	UL47	UL48	UL50	UL51	UL54	Us3	Us8A	ICP0	
Residue no. of phosphosite in HSV-1 or HSV-2 (reference)		302 (10)	288 (30)	289 (30)	371 (34)	18 (37)	162 (35)	251 (38)	253 (38)	887 (39)	889 (24)	111 (9)	77 (40)	375 (36)	187 (3)	184 (41)	114 (42)	147 (43)	61 (44)	224 (45)	226 (45)
Virus	HSV-1	S	S	S	Y	S	Y	P	G	T	Y	T	S	S	S	S	S	S	S	S	T
	BMV	S	P	P	Y	D	Y	T	P	K	Y	–	M	S	G	L	P	V	–	P	L
	ChHV	S	S	S	Y	S	Y	S	S	T	Y	T	S	S	S	P	P	D	D	S	A
	BV	S	S	A	Y	S	Y	R	D	P	Y	T	S	S	F	–	R	A	A	S	P
	HSV-2	S	S	S	Y	S	Y	S	S	A	Y	T	S	S	S	P	A	D	D	S	T
	HVA-1	S	P	D	Y	E	Y	D	G	–	R	Q	T	S	A	A	H	E	NA	L	P
	HVP-2	S	A	A	Y	S	Y	R	D	P	Y	S	A	S	F	–	R	G	R	S	P
	HVS-1	S	E	S	Y	–	Y	R	G	A	P	–	A	S	–	A	A	D	NA	N	T
	LHV-4	S	P	P	Y	E	Y	K	P	A	Y	–	V	S	D	–	R	A	NA	S	P
	MaAHV-2	S	E	D	Y	G	Y	T	K	P	Y	–	H	S	T	L	G	G	NA	S	R
	MaAHV-4	S	E	N	Y	G	Y	T	K	P	Y	–	H	S	T	L	G	V	NA	S	R
	McAHV-2	S	S	A	Y	S	Y	R	D	P	Y	T	S	S	F	–	H	A	–	S	A
	McAHV-3	S	S	A	Y	S	Y	R	D	P	Y	T	S	S	F	–	R	A	A	S	P
	MaAHV-1	S	D	N	Y	G	Y	T	K	P	Y	–	M	S	S	L	D	P	NA	S	R
	SA-8	S	A	A	Y	S	Y	R	D	P	Y	S	R	S	Y	–	R	S	–	S	P
	FBAHV-1	S	T	A	Y	S	Y	R	S	P	Y	–	R	S	F	–	R	I	R	K	L
	PLAHV	S	T	A	Y	S	Y	R	G	P	Y	–	R	S	F	–	R	I	R	–	–
Conservation (%)		100	35	24	100	59	100	12	18	12	88	35	35	100	24	5.9	5.9	12	9.1	71	18

^*a*^ Letters indicate standard one-letter amino acid codes. NA, not annotated. Gray shading indicates serine, threonine, and tyrosine residues. – indicates a gap in the alignment.

Notably, phosphorylation sites of all residue types are significantly less conserved than their respective total residue pools within the genus *Simplexvirus* (Fig. 1A), which contrasts with the trend reported in vertebrates (46, 47). These observations underscore the distinct evolutionary signatures of phospho-regulation in *Simplexvirus* species. One possible explanation for this discrepancy is that, unlike in vertebrates, *Simplexvirus* species may exploit the evolution of phospho-regulation to drive evolutionary diversification that enhances viral fitness. This interpretation is consistent with our previous observation that the acquisition of phosphorylation motifs in UL7 homologs targeted by the viral protein kinase Us3 coincides with *Simplexvirus* diversification (30).

Our results show that the phosphomimetic mutation at UL7 Tyr-89 exhibited phenotypes similar to those of the UL7 null mutation in all parameters tested in this study, suggesting that phosphorylation at UL7 Tyr-89 can function as an inhibitory switch regulating HSV-1 infection. Notably, while the phosphomimetic mutation resulted in cytoplasmic accumulation of unenveloped or partially enveloped capsids and reduced cytoplasmic and extracellular enveloped virions at levels comparable to those observed with the UL7 null mutation, it caused a significantly smaller reduction in viral replication in cultured cells. These observations suggest that phosphorylation at UL7 Tyr-89 selectively downregulates final envelopment while sparing other UL7 functions required for efficient viral replication. We should note, however, that the interpretations described above rely largely on the mutational analyses of UL7 Tyr-89. It could be argued that the observed phenotypes with amino acid substitutions at UL7 Tyr-89, especially glutamic acid substitution, are solely attributable to mutation-specific structural artifacts rather than to precluding or mimicking phosphorylation at the site, thereby inhibiting its functions. Although we cannot completely eliminate these possibilities at present, this explanation seems less likely based on our observation that the additional alanine substitution at UL7 Tyr-89 did not impair viral replication or plaque size, in contrast to the phosphomimetic mutation. Further studies will be needed to clarify these issues. Such studies include identifying the kinases and/or phosphatases responsible for regulating UL7 Tyr-89 phosphorylation and directly testing the impact of modulating Tyr-89 phosphorylation (e.g., through genetic or pharmacological inhibition of relevant enzymes). In addition, while the present study focused on cultured cells and mouse infection models, it remains to be determined how the phosphomimetic mutation at UL7 Tyr-89 influences viral replication in trigeminal ganglia or the CNS, including whether it alters transport efficiency from sensory neurons to CNS neurons. These aspects represent important directions for future study.

We observed that the non-phosphorylatable mutation at UL7 Tyr-89, which was used to evaluate the physiological relevance of this phosphorylation, significantly affected viral replication and pathogenicity in the CNS of mice. However, it had little effect on viral replication, cell-to-cell spread, or final envelopment in Vero cells, as well as on progeny virus yield in tear films and pathogenic manifestations in the eyes of mice. Two non-exclusive explanations may account for this discrepancy: (i) rapid dephosphorylation by host phosphatases and (ii) limited expression of the relevant tyrosine kinases in Vero cells and in the eyes of mice. Supporting the former, we previously reported that inhibitory tyrosine phosphorylation of HSV-2 UL13 is masked in cultured cells due to dephosphorylation by host phosphatases but remains functional *in vivo* (35). The latter possibility is also plausible, as several host tyrosine kinases are known to be expressed in murine CNS but are absent in common cell culture systems and in peripheral tissues of mice (48). These observations suggest that phosphorylation at UL7 Tyr-89 exerts tissue-specific regulatory effects, predominantly acting with functional relevance specifically in the CNS. Interestingly, the recombinant virus carrying the non-phosphorylatable mutation at UL7 Tyr-89, despite lacking the inhibitory phosphorylation at this residue, still displayed lower viral replication and pathogenicity in the CNS of mice than its repaired virus. These observations suggest that dysregulated UL7 activity resulting from the absence of phosphorylation at Tyr-89 may impair viral fitness in the CNS. Thus, precise regulation of UL7 activity through appropriately timed Tyr-89 phosphorylation appears important for optimizing viral fitness, particularly in the CNS. A similar observation has been reported for the PA-X protein of influenza A virus (49), which degrades host cellular mRNAs to shut off host protein synthesis and contributes to viral replication *in vivo* (49). PA-X variants with enhanced host shut-off activity degrade cellular mRNAs more efficiently but exhibit reduced replication efficiency *in vivo* (50). Collectively, these observations underline the relevance of fine-tuned regulation of viral protein activity to ensure effective infection in distinct tissue environments.

Albecka et al. (22) previously reported that UL7 interacts with UL51 in HSV-1-infected cells and that null mutations in both genes significantly impair viral replication to levels comparable to those observed with either single null mutation. They also showed that the double null mutation leads to the accumulation of unenveloped and partially enveloped virions in the cytoplasm, suggesting a cooperative role for UL7 and UL51 in final envelopment (22). However, their analysis of virion morphogenesis was limited to the double null mutation, leaving it unclear whether UL7 alone, UL51 alone, or both proteins are required for this process. The present study fills this gap by providing direct evidence that UL7 itself is required for efficient final envelopment. Specifically, we demonstrated that the UL7 null mutation impairs final envelopment, as evidenced by the cytoplasmic accumulation of unenveloped or partially enveloped capsids and reduced cytoplasmic and extracellular enveloped virions, without affecting the accumulation level and localization of UL51. Taken together with our earlier report (41) and that of Albecka et al. (22), which showed that the UL51 null mutation and the UL7/UL51 double null mutation exhibit similar phenotypes to the UL7 null mutation in HSV-1 virion morphogenesis, respectively, our results support a model in which UL7 promotes HSV-1 final envelopment in conjunction with UL51, thereby contributing to efficient viral replication. This conclusion is consistent with studies in other herpesviruses, suggesting that UL7 homologs contribute to secondary envelopment, including pseudorabies virus UL7 (51) and the human cytomegalovirus UL7 homolog UL103 (52).

## MATERIALS AND METHODS

### Cells and viruses

Simian kidney epithelial Vero cells, human retinal pigment epithelial ARPE-19 cells (53), Plat-GP cells derived from 293T cells and based on a murine leukemia virus packaging system, rabbit skin cells, and the HSV-1 wild-type strain HSV-1(F) were described previously (54, 55).

### Plasmids

pRetroX-TRE3G-UL6o, used to generate stable Vero cells with tetracycline-inducible UL6 expression, was constructed by cloning the entire coding sequences of codon-optimized UL6o amplified via PCR from pUC57-UL6o, which were synthesized (GenScript) using the primers 5′-gcggatccgcaaccatgacagc-3′ and 5′-gcgaattcgcaagagcgacagcgagcg-3′, into *Bam*HI and *Eco*RI sites of pRetroX-TRE3G (TaKaRa). The synthesized DNA sequences are shown in Table 3. pMAL-UL6-P1, used to generate a fusion protein of maltose-binding protein (MBP) and a domain of UL6, was constructed by cloning the domains of UL6 (encoded by UL6 codons 307-515) amplified by PCR from pYEbac102Cre (41, 56) using the primers 5′-gcgaattccgcaagagcgacagcgagcg-3′ and 5′-gcaagctttcagtgctgaaaactgttggcga-3′ into pMAL-c (New England Biolabs) in-frame with MBP.

**TABLE 3:** Summary of synthesized plasmids

Constructed plasmid	Synthesized DNA sequence^*a*^
pRetroX-TRE3G-UL6o	GGATCCgcaaccatgacagcaccaaggtctagggcacctaccacaagggcaagaggcgataccgaggccctgt gcagccctgaggacggatgggtgaaggtgcacccaacccctggcacaatgctgtttagggagatcctgcacggacagctgggatacacagagggacagggcgtgtataacgtggtgcggagctccgaggccaccacaagacagctgcaggccg ccatcttccacgccctgctgaatgccaccacatacagggatctggaggcagactggctgggacacgtggcagcaaggggactgcagccacagcgcctggtgcggagatataggaacgcaagggaggcagatatcgcaggagtggccgagagagtg ttcgacacctggaggaatacactgcgcaccacactgctggatttcgcccacggcctggtggcatgctttgcaccaggaggaccatccggcccttctagcttccctaagtacatcgactggctgacctgtctgggcctggtgccaatcctgaggaagaggca ggagggaggagtgacacagggactgagggcctttctgaagcagcaccccctgacccgccagctggccacagtggcagaggcagcagagagggcaggacctggcttctttgagctggccctggccttcgattccaccagggtggccgattacgacag agtgtatatctactataaccacaggaggggcgattggctggtgagggaccccatctctggacagaggggagagtgcctggtgctgtggccacctctgtggaccggcgataggctggtgttcgactcccctgtgcagagactgtttccagagatcgtggcctg ccactctctgagggagcacgcacacgtgtgcaggctgagaaacaccgccagcgtgaaggtgctgctgggcaggaagagcgactccgagaggggagtggcaggagcagcaagggtggtgaataaggtgctgggcgaggacgatgagacaaaggc aggcagcgccgcatccaggctggtgagactgatcatcaacatgaagggcatgcggcacgtgggcgatatcaatgacaccgtgagagcatacctggatgaggcaggaggacacctgatcgatgcaccagcagtggacggcacactgcctggatttgg caagggaggaaactctaggggcagcgccggacaggaccagggaggaagggcaccccagctgcggcaggccttcagaaccgccgtggtgaacaatatcaacggcgtgctggagggctatatcaacaatctgtttggcacaatcgagaggctgagg gagaccaatgcaggactggcaacacagctgcaggagagggatagagagctgcggagagccacctccggcgccctggagaggcagcagagggcagcagacctggcagcagagtccgtgacaggaggatgtggctctaggcctgcaggagcaga tctgctgagagcagattacgacatcatcgacgtgtctaagagcatggacgatgacacctatgtggccaatagcttccagcacccatacatcccctcctatgcccaggacctggagcggctgagcagactgtgggagcacgagctggtgaggtgctttaag atcctgtgccaccgcaacaatcagggccaggagacctccatctcttactcctctggagcaatcgcagccttcgtggcaccatattttgagagcgtgctgagggcaccaagggtgggagcacccatcaccggctccgatgtgatcctgggagaggaggagctg tgggacgccgtgttcaagaagacacggctgcagacctacctgacagatatcgccgccctgtttgtggcagacgtgcagcacgccgccctgccaccccctccatctcccgtgggcgccgatttccggcctggagcaagcccaaggggccgcagccggtc cagatctccaggacggaccgcaagaggagcaccagaccagggaggaggaatcggacacagagatggcaggcgcgacggccggagatgaGAATTC

^*a*^ Restriction enzyme sites are indicated in uppercase letters. Kozak sequences are underlined. ORF sequences are shown without additional formatting.

### Establishment of stable Vero cells with tetracycline-inducible UL6 expression

Vero cells were transduced with the supernatants of Plat-GP cells co-transfected with pMDG (57) and pRetroX-Tet3G (TaKaRa), selected with 1 mg/mL G418 (Wako), and further transduced with supernatants of Plat-GP cells co-transfected with pMDG and pRetroX-TRE3G-UL6o. For tetracycline-inducible (TetON) UL6o expression, after double selection with 5 mg/mL puromycin (Sigma) and 1 mg/mL G418, resistant cells were designated UL6o-TetON-Vero cells.

### Production and purification of GST and MBP fusion proteins

MBP-UL6 (where MBP is maltose-binding protein) was expressed in *Escherichia coli* BL21 Star (DE3) (Thermo Fisher Scientific) transformed with pMAL-UL6-P1 and purified using amylose resin (New England BioLabs), as described previously (58).

### Antibodies

Commercial antibodies used in this study were mouse monoclonal antibodies to α tubulin (DM1A; Sigma), Flag (M2; Sigma), and ICP27 (H1142; Santa Cruz Biotechnology). Rabbit polyclonal antibodies to UL7, UL18 (VP23), UL48 (VP16), UL50 (vdUTPase), and UL51 were previously described (30, 41, 59). To generate mouse polyclonal antibodies against HSV-1 UL6, BALB/c mice were immunized once with purified MBP-UL6-P1 using TiterMax Gold (TiterMax USA, Inc.) as an adjuvant. Sera from immunized mice were used as sources of mouse polyclonal antibodies against UL6.

### Construction of recombinant HSV-1 viruses

Recombinant viruses YK440 (UL6Y231F), YK441 (UL6Y231E), YK442 (UL6Y234F), YK443 (UL6Y234E), YK480 (UL7Y89F), YK482 (UL7Y89E), and YK484 (ΔUL7) were generated by a two-step Red-mediated mutagenesis procedure (43, 60) using *E. coli* GS1783 containing pYEbac102Cre, a full-length infectious HSV-1(F) clone (41, 56), pEP-KanS (60), and the primers listed in Table 4. To construct recombinant viruses YK486 (3xFlag-UL7Y89F) and YK487 (3xFlag-UL7Y89E), we first constructed a version of the HSV-1 genome encoding UL7 with a 3xFlag tag inserted between amino acids 238 and 239 (3xFlag-UL7) in *E. coli* GS1783 harboring pYEbac102Cre, using the two-step Red-mediated mutagenesis with pEP-KanS and the primers listed in Table 4. Subsequently, Tyr-89 in UL7 was substituted with phenylalanine or glutamic acid by the same procedure, again using pEP-KanS and the corresponding primers listed in Table 4, to generate the Y89F and Y89E variants, respectively. Recombinant viruses YK444 (UL6Y234E-repair), YK481 (UL7Y89F-repair), YK483 (UL7Y89E-repair), YK485 (ΔUL7-repair), and YK488 (3xFlag-UL7Y89E-repair), in which the mutations in UL7 were repaired (Fig. 3), were generated by a two-step Red-mediated mutagenesis procedure using *E. coli* GS1783 containing the UL6Y234E, UL7Y89F, UL7Y89E, ΔUL7, and 3xFlag-UL7 genomes, pEP-KanS, and primers listed in Table 4. Recombinant viruses YK445 (UL6E234A) and YK489 (UL7E89A), in which alanine substitutions were introduced as control mutations, were generated by a two-step Red-mediated mutagenesis procedure using *E. coli* GS1783 containing the UL6Y234E and UL7Y89E genomes, pBS-Venus-KanS, and primers listed in Table 4. The genotype of each recombinant virus was confirmed by PCR and/or sequencing.

**TABLE 4:** Oligonucleotide sequences for the construction of recombinant viruses.

Recombinant virus	Oligonucleotide sequence (5′−3′)	DNA template	*E. coli* GS1873 containing HSV-1 BAC
UL6Y231F	5′-GGCCTTCGACTCCACGCGCGTGGCGGACTACGACCGCGTGTTTATTTA CTACAACCACCGAGGATGACGACGATAAGTAGGG-3′	pEP-KanS (60)	*E. coli* GS1783 containing the pYEbac102Cre (40, 56)
	5′-CTCGCACGAGCCAGTCGCCCCGGCGGTGGTTGTAGTAAATAAACACG CGGTCGTAGTCCGCAACCAATTAACCAATTCTGATTAG-3′		
UL6Y231E	5′-GGCCTTCGACTCCACGCGCGTGGCGGACTACGACCGCGTGGAAATTT ACTACAACCACCGAGGATGACGACGATAAGTAGGG-3′	pEP-KanS (60)	*E. coli* GS1783 containing the pYEbac102Cre (40, 56)
	5′-CTCGCACGAGCCAGTCGCCCCGGCGGTGGTTGTAGTAAATTTCCACG CGGTCGTAGTCCGCAACCAATTAACCAATTCTGATTAG-3′		
UL6Y234F	5′-CTCCACGCGCGTGGCGGACTACGACCGCGTGTATATTTACTTCAACC ACCGCCGGGGCGAAGGATGACGACGATAAGTAGGG-3′	pEP-KanS (60)	*E. coli* GS1783 containing the pYEbac102Cre (40, 56)
	5′-TGATGGGGTCTCGCACGAGCCAGTCGCCCCGGCGGTGGTTGAAGTAA ATATACACGCGGTCAACCAATTAACCAATTCTGATTAG-3′		
UL6Y234E	5′-CTCCACGCGCGTGGCGGACTACGACCGCGTGTATATTTACGAGAACC ACCGCCGGGGCGAAGGATGACGACGATAAGTAGGG-3′	pEP-KanS (60)	*E. coli* GS1783 containing the pYEbac102Cre (40, 56)
	5′-TGATGGGGTCTCGCACGAGCCAGTCGCCCCGGCGGTGGTTCTCGTAA ATATACACGCGGTCAACCAATTAACCAATTCTGATTAG-3′		
UL6Y234E-repair	5′-CTCCACGCGCGTGGCGGACTACGACCGCGTGTATATTTACTACAACC ACCGCCGGGGCGAAGGATGACGACGATAAGTAGGG-3′	pEP-KanS (60)	*E. coli* GS1783 containing the UL6Y234E genome (this study)
	5′-TGATGGGGTCTCGCACGAGCCAGTCGCCCCGGCGGTGGTTGTAGTAA ATATACACGCGGTCAACCAATTAACCAATTCTGATTAG-3′		
UL6E234A	5′-CTCCACGCGCGTGGCGGACTACGACCGCGTGTATATTTACGCCAACCA CCGCCGGGGCGAAGGATGACGACGATAAGTAGGG-3′	pBS-Venus-KanS (60)	*E. coli* GS1783 containing the UL6Y234E genome (this study)
	5'- TGATGGGGTCTCGCACGAGCCAGTCGCCCCGGCGGTGGTTGGCGTAA ATATACACGCGGTCAACCAATTAACCAATTCTGATTAG −3'		
UL7Y89F and 3xFlag-UL7Y89F	5′-GGACGGGAGTCCCGAGGACGCCTATGTGACGTCGGAGGATTTCTTTA AGCGCTGCTGCGGAGGATGACGACGATAAGTAGGG-3′	pEP-KanS (60)	*E. coli* GS1783 containing the pYEbac102Cre (40, 56) and 3xFlag-UL7 genome (this study)
	5′-CGAAGCCGCGATAACTGGACTGGCCGCAGCAGCGCTTAAAGAAATC CTCCGACGTCACATCAACCAATTAACCAATTCTGATTAG-3′		
UL7Y89E and 3xFlag-UL7Y89E	5′-GGACGGGAGTCCCGAGGACGCCTATGTGACGTCGGAGGATGAATTTA AGCGCTGCTGCGGAGGATGACGACGATAAGTAGGG-3′	pEP-KanS (60)	*E. coli* GS1783 containing the pYEbac102Cre (40, 56)
	5′-CGAAGCCGCGATAACTGGACTGGCCGCAGCAGCGCTTAAATTCATCC TCCGACGTCACATCAACCAATTAACCAATTCTGATTAG-3′		
ΔUL7	5′-CGCCGCGACGGCCGACGATGAGGGGTCGGCCGCCACCATCTAGTAA TGAGCCATCGCCGGGGACCGAGGATGACGACGATAAGTAGGG-3′	pEP-KanS (60)	*E. coli* GS1783 containing the pYEbac102Cre (40, 56)
	5′-CCTCGGCCGCCTCGACCAGGCTGCGGTCCCCGGCGATGGCTCATTAC TAGATGGTGGCGGCCGACCCAACCAATTAACCAATTCTGATTAG-3′		
ΔUL7-repair	5′-CGCCGCGACGGCCGACGATGAGGGGTCGGCCGCCACCATCCTCAAGC AGGCCATCGCCGGGGACCGAGGATGACGACGATAAGTAGGG-3′	pEP-KanS (60)	*E. coli* GS1783 containing the ΔUL7 genome (this study)
	5′-CCTCGGCCGCCTCGACCAGGCTGCGGTCCCCGGCGATGGCCTGCTTG AGGATGGTGGCGGCCGACCCAACCAATTAACCAATTCTGATTAG-3′		
3xFlag-UL7	5′-CCCGCCCCCCGTTCTCTCGGCCCTGTTCTGTGCCACCCCGGACTACAA AGACCATGACGGTGATTATAAAGATCATGACATCGATTACAAGGATGACGAT GACAAAACAAGCTCAGGATGACGACGATAAGTAGGG-3′	pEP-KanS (60)	*E. coli* GS1783 containing the pYEbac102Cre (40, 56)
	5′-AGCGGGGGGGCGGCCCCGGCAGCCGGAATGAGGAGCTTGTTTTGTCA TCGTCATCCTTGTAATCGATGTCATGATCTTTATAATCACCGTCATGGTCTTT GTAGTCCGGGGTGGCAACCAATTAACCAATTCTGATTAG-3′		
UL7Y89F-repair, UL7Y89E-repair, and 3xFlag-UL7Y89E-repair	5′-GGACGGGAGTCCCGAGGACGCCTATGTGACGTCGGAGGATTACTTTA AGCGCTGCTGCGGAGGATGACGACGATAAGTAGGG-3′	pEP-KanS (60)	*E. coli* GS1783 containing the UL7Y89F, UL7Y89E, and 3xFlag-UL7Y89E genomes (this study)
	5′-CGAAGCCGCGATAACTGGACTGGCCGCAGCAGCGCTTAAAGTAATCC TCCGACGTCACATCAACCAATTAACCAATTCTGATTAG-3′		
UL7E89A	5’- GGACGGGAGTCCCGAGGACGCCTATGTGACGTCGGAGGATGCCTTT AAGCGCTGCTGCGGAGGATGACGACGATAAGTAGGG-3′	pBS-Venus-KanS (61)	*E. coli* GS1783 containing the UL7Y89E genome (this study)

### Immunoblotting, immunofluorescence, and determination of plaque size

Immunoblotting and immunofluorescence assays were performed as described previously (41, 62, 63).

### Electron microscopic analysis

Vero cells infected with wild-type HSV-1(F), YK480 (UL7Y89F), YK481 (UL7Y89F-repair), YK482 (UL7Y89E), YK483 (UL7Y89E-repair), YK484 (ΔUL7), and YK485 (ΔUL7-repair) at an MOI of 3 for 18 h were examined by ultrathin-section electron microscopy as described previously (64). The number of virus particles at different morphogenetic stages was quantitated in randomly chosen cells.

### Animal studies

Three-week-old female ICR mice (Charles River) were infected intracranially with 1 × 10^3^ PFU of the indicated viruses as described previously (10, 54, 61, 65). Mice were monitored daily, and mortality occurring from 1 to 14 days post-infection was attributed to the infecting virus. To measure viral titers in the brains of infected mice, 3-week-old female ICR mice were each infected intracranially with 1 × 10^3^ PFU of each selected virus. At 3 days post-infection, the brains of the mice were harvested, and viral titers were determined on Vero cells. Five-week-old female ICR mice (Charles River) were infected ocularly with 1 × 10^5^ PFU/eye of the indicated viruses as described previously (53, 61, 66). The scoring scales for the severity of herpes stromal keratitis were described previously (53, 66). Viral titers in tear films were determined as described previously (53, 61, 66).

### Statistical analysis

Differences in the ratios of phospho-residue conservation were statistically analyzed using Welch’s one-way ANOVA, followed by two-tailed Welch’s *t*-tests to account for unequal variances and sample sizes. *P*-values were adjusted using the Benjamini–Hochberg procedure, and adjusted values (*q*) were used to define statistical significance (*q* < 0.05). For clarity and accessibility, unadjusted *P*-values are reported in figure legends and the Results section, whereas *q*-values were used to determine significance thresholds. Effect sizes were estimated as partial *w*² for omnibus tests and Hedges’ *g* for pairwise comparisons. Differences in colocalization coefficients, viral yields, and capsid counts in cell cultures were analyzed by one-way ANOVA followed by Tukey’s *post hoc* test. Viral yields from infected cells and mouse samples (brain and tear film), as well as mouse disease scores, were analyzed using unpaired two-tailed Student’s *t*-tests. Survival curves were compared using the log-rank test. A *P*-value < 0.05 was considered statistically significant.

## Data Availability

The amino acid sequences used in this study are available from the National Center for Biotechnology Information. The viruses and their corresponding accession numbers for these genome sequences are listed in Table S1. The structures of UL6 (PDB ID: 6od7) and UL7 (PDB ID: 6t5a) are available in the Protein Data Bank. Additional data supporting the findings of this study are available from the corresponding author upon reasonable request.

## SUPPLEMENTAL MATERIAL

Fig. S1 jvi.00200-26-s0001.pdf Conservation percentages of phosphorylation sites on HSV-1 proteins among members of the genus *Simplexvirus*.

Table S1 jvi.00200-26-s0002.pdf Accession numbers of viral sequences used in this study.

Table S2 jvi.00200-26-s0003.xlsx Conservation of phosphorylation sites on HSV-1 proteins among members of the genus *Simplexvirus*.

ASM does not own the copyrights to Supplemental Material that may be linked to, or accessed through, an article. The authors have granted ASM a non-exclusive, world-wide license to publish the Supplemental Material files. Please contact the corresponding author directly for reuse.
