# Supplementary material for: Evolution-guided prioritization identifies a tissue-specific phosphorylation switch on herpes simplex virus 1 UL7 regulating viral replication and pathogenicity
Source: J Virol. 2026 Apr 30;100(5):e00200-26. doi: 10.1128/jvi.00200-26 (PMC13185574; doi:10.1128/jvi.00200-26)
Supplement: Fig. S1 — Conservation percentages of phosphorylation sites on HSV-1 proteins among members of the genus Simplexvirus. [file jvi.00200-26-s0001.pdf]

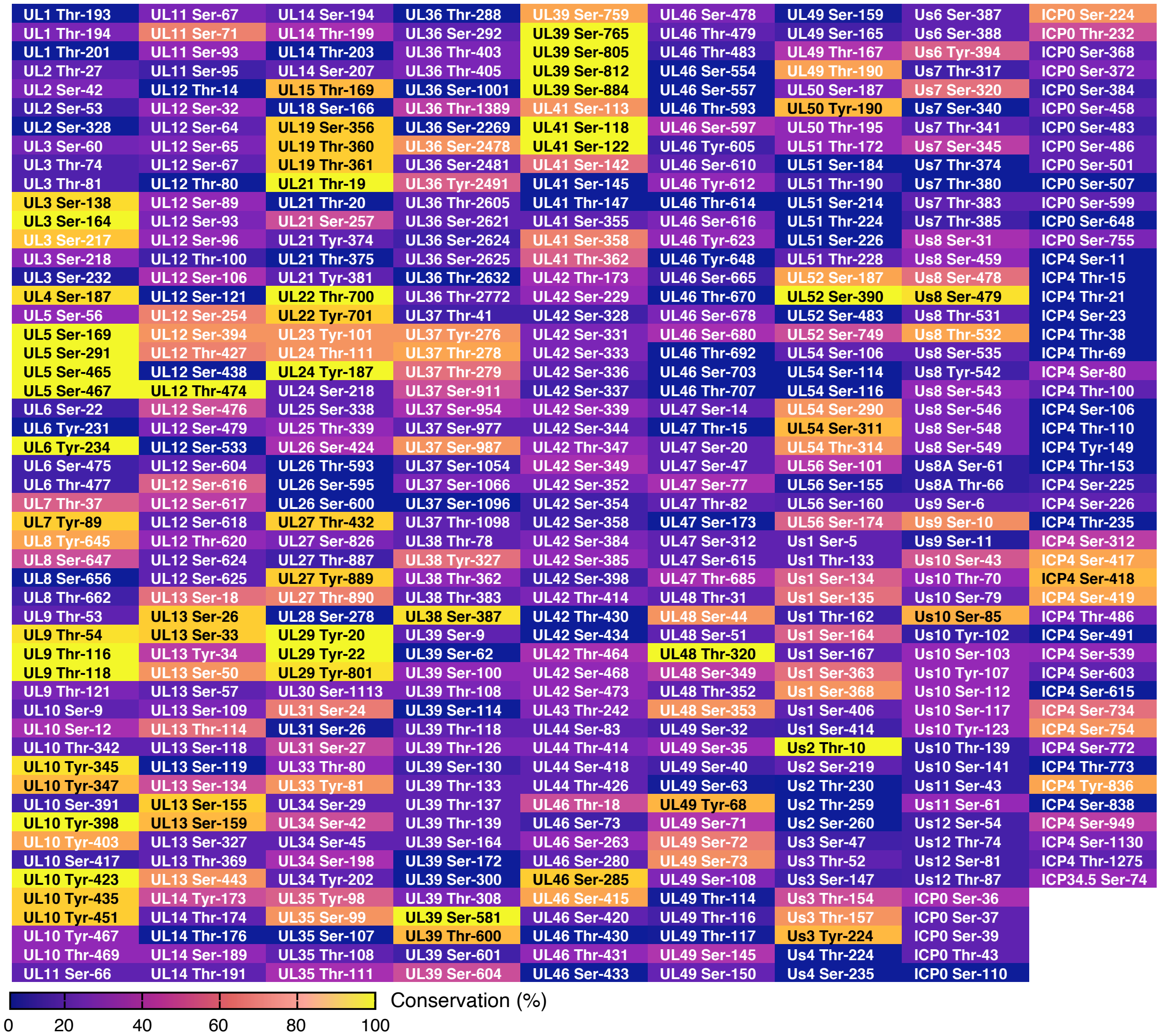

Supplemental figure 1. Conservation percentages of phosphorylation sites on HSV-1 proteins among members of the genus Simplexvirus. Heatmap showing the conservation ratio of phosphorylation sites on HSV-1 viral proteins, reported through phosphoproteome analysis of HSV-1–infected human foreskin fibroblasts (4), among members of the genus Simplexvirus. Color scale indicates conservation percentages, with values increasing from purple (low) to yellow (high). Residues with ≥88% conservation are labeled in black.

Supplemental figure 1. A. Kato and T. Tannaka et al.
