# Supplementary material for: Evolution-guided prioritization identifies a tissue-specific phosphorylation switch on herpes simplex virus 1 UL7 regulating viral replication and pathogenicity
Source: J Virol. 2026 Apr 30;100(5):e00200-26. doi: 10.1128/jvi.00200-26 (PMC13185574; doi:10.1128/jvi.00200-26)
Supplement: Table S1 — Accession numbers of viral sequences used in this study. [file jvi.00200-26-s0002.pdf]

Table S1. Accession numbers of viruses sequences using in this study.

Virus	Accession Number	Link
Human alphaherpesvirus 1 (HSV-1)	JN555585	https://www.ncbi.nlm.nih.gov/nuccore/JN555585
Bovine alphaherpesvirus 2 (BMV)	MT862163	https://www.ncbi.nlm.nih.gov/nuccore/MT862163
Panine alphaherpesvirus 3 (ChHV)	JQ360576	https://www.ncbi.nlm.nih.gov/nuccore/JQ360576
Macacine alphaherpesvirus 1 (BV)	AF533768	https://www.ncbi.nlm.nih.gov/nuccore/AF533768
Human alphaherpesvirus 2 (HSV-2)	JN561323	https://www.ncbi.nlm.nih.gov/nuccore/JN561323
Ateline alphaherpesvirus 1 (HVA-1)	KY38563	https://www.ncbi.nlm.nih.gov/nuccore/KY385637
Papiine alphaherpesvirus 2 (HPV-2)	DQ149153	https://www.ncbi.nlm.nih.gov/nuccore/DQ149153
Saimiriine alphaherpesvirus 1 (HVS-1)	HM625781	https://www.ncbi.nlm.nih.gov/nuccore/HM625781
Leporid alphaherpesvirus 4 (LHV-4)	JQ596859	https://www.ncbi.nlm.nih.gov/nuccore/JQ596859
Macropodid alphaherpesvirus 2 (MaAHV-2)	MT900475	https://www.ncbi.nlm.nih.gov/nuccore/MT900475
Macropodid alphaherpesvirus 4 (MaAHV-4)	MT900474	https://www.ncbi.nlm.nih.gov/nuccore/MT900474
Macacine alphaherpesvirus 2 (McAHV-2)	KY628968	https://www.ncbi.nlm.nih.gov/nuccore/KY628968
Macacine alphaherpesvirus 3 (McAHV-3)	KY628970	https://www.ncbi.nlm.nih.gov/nuccore/KY628970
Macropodid alphaherpesvirus 1 (MaAHV-1)	KT594769	https://www.ncbi.nlm.nih.gov/nuccore/KT594769
Cercopithecine alphaherpesvirus 2 (SA-8)	AY714813	https://www.ncbi.nlm.nih.gov/nuccore/AY714813
Teropodid alphaherpesvirus 1 (FBAHV-1)	AB825953	https://www.ncbi.nlm.nih.gov/nuccore/AB825953
Pteropodid alphaherpesvirus 2 (PLAHV)	LC492974	https://www.ncbi.nlm.nih.gov/nuccore/LC492974
